# PHPT1/Ocnus negatively regulates the JAK/STAT pathway to control cell survival

**DOI:** 10.1016/j.isci.2026.117537

**Published:** 2026-09-17

**Authors:** Qian Wang, Yue Ren, Meng-Yan Chen, Bin Mao, Xuan Guo, Jian Wan, Ke Liu, Jing-Jing Tong, Yu-Feng Wang

**Affiliations:** 1 Key Laboratory of Pesticide & Chemical Biology of Ministry of Education, Hubei Key Laboratory of Genetic Regulation and Integrative Biology, School of Life Sciences, Central China Normal University, Wuhan 430079, China; 2 School of Biology and Agricultural Resources, Huanggang Normal University, Huanggang 438000, China; 3 Life Science Institute, Jinzhou Medical University, Jinzhou 121001, China

**Keywords:** PHPT1/*ocnus*, *Drosophila melanogaster*, spermatogenesis, JAK/STAT signaling pathway, apoptosis

## Abstract

The JAK/STAT signaling pathway is a highly conserved regulator essential for animal development. Here, we show that the *Drosophila* phosphohistidine phosphatase 1, PHPT1/Ocnus, is required for cell survival by regulating JAK/STAT signaling. Knockdown of *ocnus* (*ocn*) leads to apoptosis of germ cells during the transit-amplifying divisions of spermatogonia in the second instar larvae. In *Drosophila* S2 cells, *ocn* overexpression reduces phosphorylated STAT (pSTAT) levels, limits its nuclear accumulation, and alters the expression of STAT target genes. Mechanistically, Ocn directly dephosphorylates Stat92E at the conserved tyrosine residue Y711. Consistently, genetic inhibition of JAK/STAT signaling via Socs36E overexpression partially rescues male sterility caused by *ocn* knockdown. Moreover, human PHPT1 suppresses nuclear accumulation of pSTAT in HeLa cells, indicating evolutionary conservation. Together, our findings identify PHPT1/Ocnus as a conserved negative regulator of JAK/STAT signaling and may provide a potential target for therapeutic intervention in the treatment of STAT-related diseases, including cancer.

## Highlights

• Knockdown of *Drosophila PHPT1* (*ocnus*) causes germ cell loss via apoptosis
• Ocnus directly dephosphorylates STAT on the conserved Y711 of Stat92E
• *ocnus* knockdown induces apoptosis during mitosis by stimulating JAK/STAT signaling
• Human PHPT1 plays a similar role in negatively regulating JAK/STAT signaling

## Graphical abstract

**Figure:**
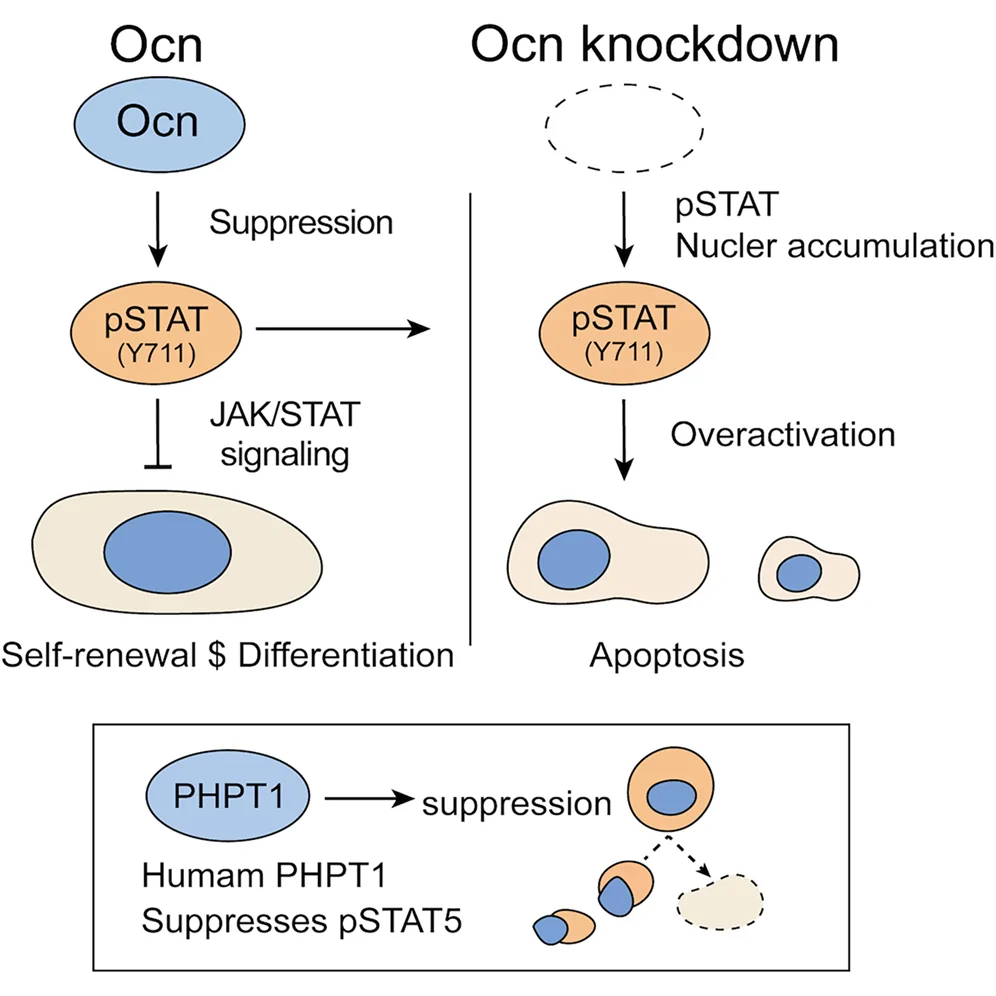

## Teaser

Protein; Molecular biology; Developmental biology

## Introduction

The Janus kinase/signal transducer and activator of transcription (JAK/STAT) signaling pathway exhibits both structural and functional conservation from invertebrates to vertebrates.^1^ This pathway plays an essential role during development, including gametogenesis, maintenance and differentiation of stem cells, cell proliferation, and cell death.^2^^,^^3^^,^^4^^,^^5^^,^^6^ For example, at the anterior tip of *Drosophila* testis, hub cells produce the cytokine-like ligand unpaired (Upd). This ligand activates the JAK/STAT pathway, which is crucial for the maintenance and self-renewal of two types of stem cells required for male fertility: germline stem cells (GSCs, source of germ cells, initially differentiate into gonialblasts [GBs]) and somatic cyst stem cells (CySCs, source of cyst cells that encapsulate germ cells to form cysts and support spermatogenesis).^4^^,^^7^^,^^8^ Any defects causing insufficient or excessive activity of this pathway can induce severe diseases, such as cancer.^2^^,^^3^^,^^9^ The JAK/STAT pathway is a key signal that persists through the level of nuclear accumulation of phosphorylated STAT (pSTAT).^1^^,^^10^ In resting cells, the majority of STAT proteins are primarily localized within the cytoplasm. However, upon stimulation by cytokines/Upd, the tyrosine-phosphorylated dimerized STATs display a notable nuclear accumulation.^4^^,^^11^^,^^12^

During *Drosophila* spermatogenesis, GSCs undergo asymmetric divisions, producing new GSCs and GBs. One GB undergoes four cycles of mitotic transit-amplification, resulting in 16 interconnected spermatogonia. They are encapsulated by two cyst cells, forming a cyst. The spermatogonia then continue to grow, becoming larger spermatocytes. Following two rounds of meiosis, these spermatocytes develop into 64 haploid spermatids, which subsequently differentiate into mature sperm.^13^^,^^14^ *ocnus* (*ocn*) is a novel gene that encodes a protein highly expressed in fly testes.^15^ Ocn has been predicted to enable protein histidine phosphatase activity and to be orthologous to human PHPT1 (phosphohistidine phosphatase 1) (flybase.org). PHPT1 has also been demonstrated to be highly expressed in the testes of mice and humans.^16^ Our previous studies have shown that the depletion of *ocn* led to much smaller testes with no germ cells and the expansion of hub signal in 1-day-old adult flies.^17^ This small testis is reminiscent of the phenotypes involved in defects in JAK/STAT signaling.^18^ Quantitative proteomics and phosphoproteomics revealed that after *ocn-*knockdown (KD) the protein level of Stat92E was not significantly different, while the phosphorylated Stat92E was significantly elevated to 1.45-fold in the abdomen of *Drosophila melanogaster* (*D. melanogaster*).^19^ Considering that JAK/STAT signaling is critical in fly testes for GSC maintenance and differentiation, and *ocn-*KD in germline induced the loss of germ cells in the testis of adult flies, we infer that *ocn* may regulate male germ cell development through the JAK/STAT pathway.

Here, we aimed to explore whether and how *ocn* regulates male germ cell development through the JAK/STAT signaling pathway. We found that *ocn-*KD in early germline led to the loss of germ cells during transit-amplification of spermatogonia in the 2^nd^ instar larval stages through apoptosis. Additionally, *ocn* negatively regulated the expressions of STAT target genes through mediating the nuclear/cytoplasmic ratio of pSTAT through dephosphorylating pY711 of *Drosophila* Stat92E. Human PHPT1 played a similar role in regulating the distribution of pSTAT within HeLa cells. These suggest that PHPT1/Ocn may regulate cell survival via negatively modulating the JAK/STAT signaling pathway. Given that JAK/STAT signaling regulates numerous developmental processes, our findings may provide a previously unappreciated therapeutic strategy for targeting STAT-mediated diseases.

## Results

### *ocn*-KD does not affect the specification and migration of PGCs in the developing embryo

**Figure 1:**
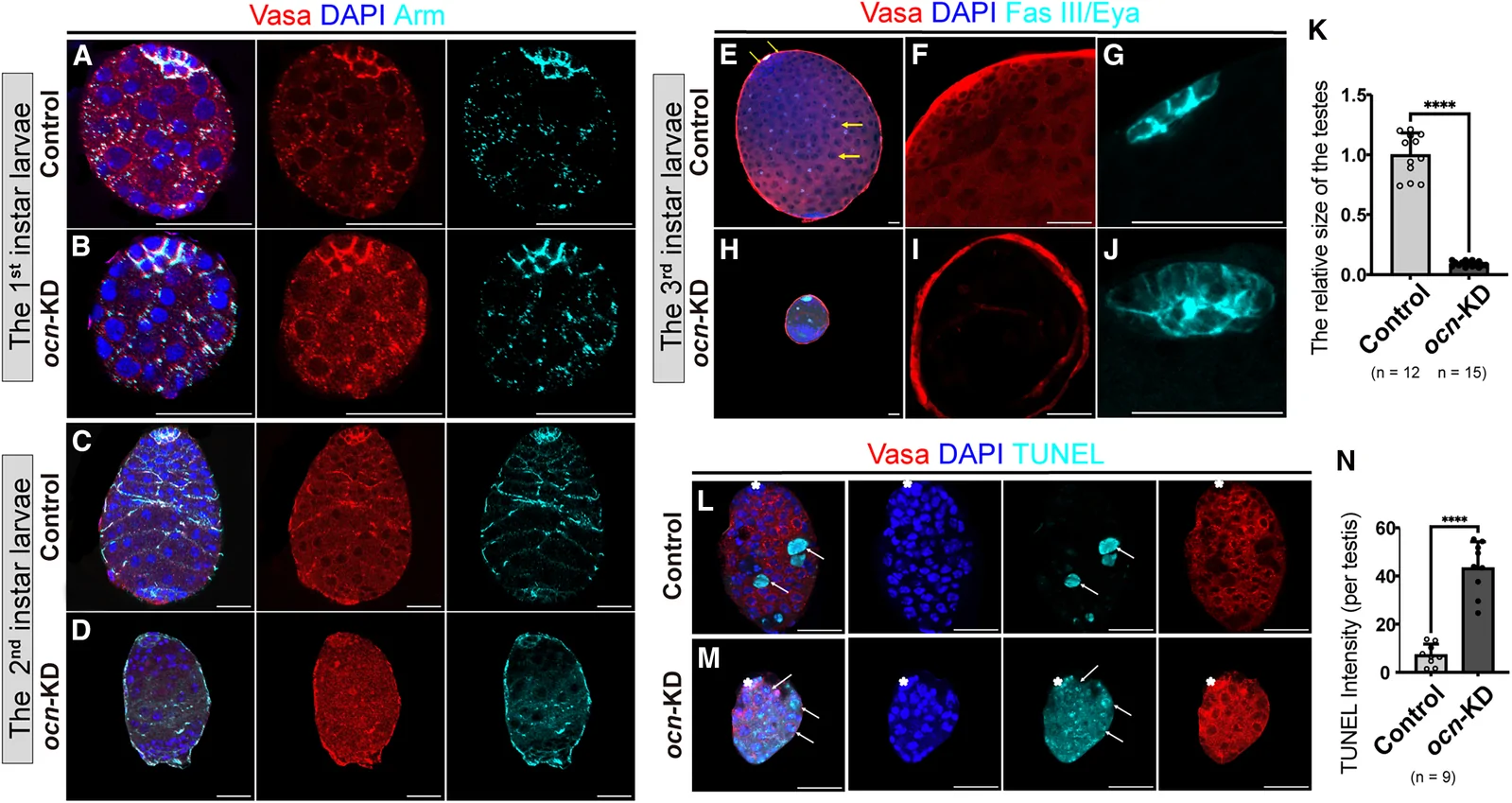
*ocn* knockdown resulted in the loss of male germ cells during larval stages through apoptosis Anti-Vasa staining (red) labels germ cells, and DAPI (blue) marks nuclei. Hub cells and the somatic support cell cytoplasmic extensions surrounding the differentiating germ cells (cysts) are labeled by anti-Arm staining (cyan, A–D). Hub cells are marked by anti-Fas III staining (cyan, E, G, H, and J). Cyst cells are marked by anti-Eya staining (cyan, E and H). (A and B) The 1^st^ instar larval testes. (A) Control (*nosGal4* > *w*^*-*^); (B) *ocn* knockdown (*ocn*-KD; *nosGal4* > *ocn-RNAi*). Representative images are shown from a single experiment; a total of 15 testes were examined. (C and D) The 2^nd^ instar larval testes. (C) Control; (D) *ocn*-KD. Representative images are shown from three independent experiments; a total of 25 testes were examined. (E–J) The 3^rd^ instar larval testes. (E and G) Control; (H–J) *ocn*-KD. (F and G) Enlarged views of the anterior region of control testis in E. (I and J) Enlarged views of the anterior region of *ocn*-KD testis in (H). Representative images are shown from three independent experiments; a total of 20 testes were examined. (K) The relative area of the testes in the 3^rd^ instar larvae of the control and *ocn*-KD flies. (L and M) TUNEL (cyan) labels the apoptotic cells in the 2^nd^ instar larval stage. The asterisk indicates hub position. (N) The TUNEL intensity per testis in the control and *ocn*-KD larval testes. A total of 17 testes from three independent experiments were analyzed. *n* represents the number of biologically independent testes. Data are presented as mean ± SEM, and significant difference between two groups was determined by the Student’s *t* test and indicated by asterisks: ∗∗∗∗*p* < 0.0001. Scale bars, 25 μm.

In *D. melanogaster*, germ cells are generated in the syncytial embryo when nuclei migrate to the posterior germplasm and cellularize to form primordial germ cells (PGCs).^20^ During gastrulation, PGCs are passively dragged into the embryo and then they actively migrate and ultimately settle in the embryonic gonads at stage 13.^21^ Our previous work revealed that *ocn-*KD induced much smaller testes devoid of germ cells in 1-day-old flies.^17^ The specificity was already validated using two independent RNAi lines and an overexpression rescue line, confirming that the observed defects are not due to off target effects.^19^ To determine the time of germ cell loss, we stained the developing embryos using Vasa (germ cell marker) and Fas III (hub cell marker) antibodies. We observed that by stage 4, the PGCs were normally formed at the posterior end of the *ocn-*KD embryo (Figures S1A, S1B, S1A′, and S1B′). During gastrulation, these PGCs were initially displaced into the midgut and subsequently passed through the gut in a manner identical to that observed in controls (Figures S1C, S1D, S1C′, and S1D′). They then migrated to the posterior region as expected and were encapsulated by mesodermal cells of the gonad (Figures S1E, S1F, S1E′, and S1F′). Consistent with control embryos, Vasa signals were clearly present in germ cells and the Fas III was properly restricted at the anterior tip of the male gonad in *ocn-*KD embryos (Figures S1G, S1H, S1G′, and S1H′). These results suggest that, unlike the later larval phenotype described in Figure 1, the KD of *ocn* did not impede the formation and migration of PGCs during embryonic development.

### *ocn*-KD results in the loss of germ cells during larval stages through apoptosis

To continue tracking the development of male germ cells in post-embryonic stages, we examined the larval testes. We observed that in the 1^st^ instar larval stage, the testes of *ocn*-KD flies (Figure 1B) were similar in size to those of control flies (Figure 1A), and anti-Vasa staining showed the presence of early germ cells in both *ocn*-KD and control testes. By the 2^nd^ instar larvae, the control testes (Figure 1C) were observed with a few cysts (enclosed by Arm signals) containing 2-16 germ cells, whereas *ocn*-KD testes (Figure 1D) were slightly smaller and showed fewer vasa-positive cells and smaller cysts with fewer than two germ cells. Surprisingly, in the 3^rd^ instar larval stage, *ocn*-KD testes were dramatically smaller (Figures 1E and 1H), only 0.09-fold relative to the area of controls (approximately an 11-fold reduction compared to control) (Figure 1K). These small testes lacked vasa-positive germ cells (Figures 1H and 1I), while the control testes contained small GSCs and spermatogonia (small arrows) at the apex, diffusely bigger spermatocytes (bigger arrows) distal from the apex, and Eya-positive cyst cells (white dots) (Figure 1E). Additionally, hub cells in *ocn*-KD testes were extended (Figure 1J), whereas in control testes (Figures 1E and 1G), they were confined to the apical tips.

To investigate whether apoptosis contributed to the loss of germ cells in the 2^nd^ instar larval testes, we performed TUNEL staining. Surprisingly, the TUNEL signal was notably stronger in *ocn*-KD testes (Figure 1M) compared to control testes (Figure 1L). While a few cysts in control testes exhibited TUNEL positivity (white arrows, green channel), indicating occasional apoptotic stimuli,^22^ nearly all germ cells in *ocn*-KD testes showed apoptotic signal (white arrows denote representative apoptotic germ cells). The TUNEL fluorescence intensity in *ocn*-KD testes was approximately 5.96-fold higher than in control testes (Figure 1N). Collectively, these findings suggest that the loss of germ cells in *ocn*-KD testes occurs via apoptosis during spermatogonial transit-amplification in the 2^nd^ instar larvae.

### Ocn is involved in JAK/STAT signaling pathway

**Figure 2:**
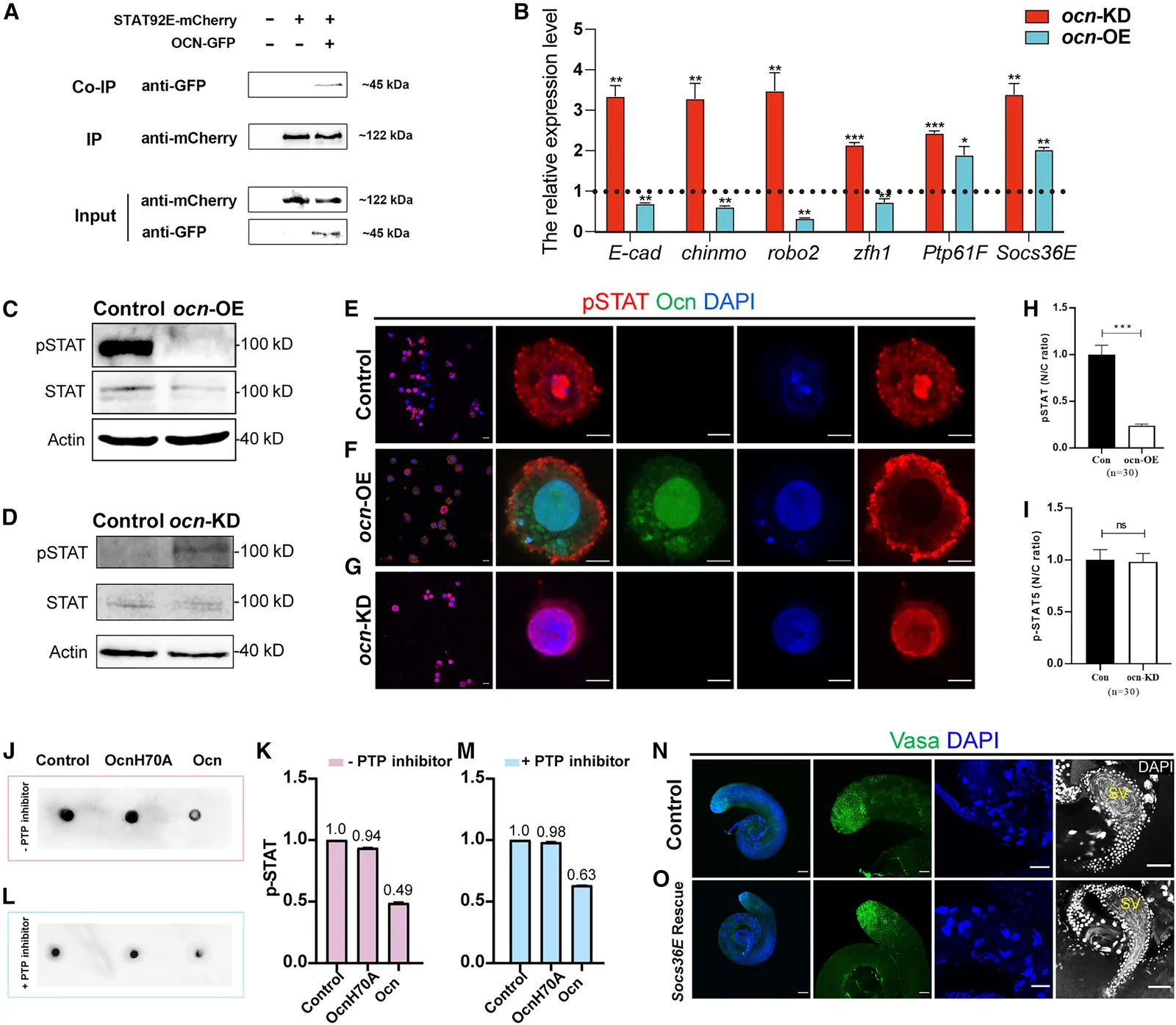
Ocn negatively regulates the JAK/STAT signaling pathway through dephosphorylating Stat92E (A) CoIP reveals the interaction of Ocn with Stat92E. Representative images are shown from two independent repeat experiments. (B) Ocn is involved in the expression of Stat92E target genes. Data were obtained from at least three biological replicates and are presented as mean ± SEM. Student’s *t* test, ∗*p* < 0.05; ∗∗*p* < 0.01; ∗∗∗*p* < 0.001. (C) Overexpression of *ocn* in S2 cells led to a decrease in phosphorylated Stat92E (pSTAT) levels. Representative images are shown from two independent repeat experiments. (D) Knockdown of *ocn* in fly testes resulted in an increase in pSTAT levels. Actin was used as a loading control. Representative images are shown from two independent repeat experiments. (E–I) Ocn affects the nuclear accumulation of pSTAT. (E) S2 cells transfected with a control construct. (F) S2 cells transfected with *ocn-GFP* plasmid (*ocn*-OE). (G) S2 cells transfected with a construct containing *ocn*-siRNA (*ocn*-KD). Scale bars: 10 μm (the first column for E, F, and G); 5 μm (other columns for E, F, and G). (H and I) The nucleoplasmic fluorescence signal intensity of pSTAT in the *ocn*-OE (G) or *ocn*-KD (I) cells relative to the control (Con) cells. *n* represents the number of S2 cells from two independent repeat experiments. Data are shown as mean ± SEM. Student’s *t* test, ∗∗∗*p* < 0.001; ns, not significant. (J–M) Dot blot analysis showing the dephosphorylation activity of Ocn toward the phosphotyrosine residue of STAT (corresponding to the Y711 site of Stat92E), with reactions conducted under conditions either in the presence (L and M) or absence (J and K) of protein-tyrosine phosphatase (PTP) inhibitor. The relative dot blot signal intensities of pSTAT from Ocn- or OcnH70A-treated groups compared to controls were determined under reaction conditions without (K) and with (M) PTP inhibitor. For (J and L) representative images are shown from four independent repeat experiments. Data in (K and M) represent the relative values of the means obtained from four independent repeat experiments. (N and O) The overexpression of *Socs36E* partially rescues the male sterility caused by *ocn* knockdown in early germline of *D. melanogaster*. (N) Testis from control flies. (O) Testis from *ocn* knockdown and simultaneous *Socs36E* overexpression (*Socs36E* Rescue: *nosGal4*>*ocn-RNAi*; *UAS-Socs36E*) flies, which contain Vasa-positive cells, some spermatid nuclear bundles in the base, and a few mature sperm with needle-like nuclei in the seminal vesicle (SV). A total of 10 fly testes from two independent experiments were analyzed. Scale bars: 100 μm (the first column for N and O); 50 μm (the second column for N and O); and 25 μm (other columns for N and O).

The phenotype of small size of *ocn*-KD testis looked like the defects observed in JAK/STAT signaling.^18^ Our previous phosphoproteomics analyses revealed that the level of phosphorylated Stat92E was significantly increased following *ocn-*KD in fly testes.^19^ Therefore, we hypothesized that *ocn* may function in germ cell development through JAK/STAT signaling pathway in fly testes. Considering the complex composition of testicular cells, we tested this hypothesis using *Drosophila* S2 cells. Co-immunoprecipitation, CoIP analyses showed a potential direct interaction between Ocn protein and Stat92E (Figure 2A).

In *Drosophila*, several Stat92E target genes including *E-cad*, *chinmo*, *robo2*, *zfh1*, *Ptp61F*, and *Socs36E* have been identified.^1^^,^^4^ We then examined the expression of these target genes in *Drosophila* S2 cells. We knocked down *ocn* and overexpressed *ocn* in S2 cells, respectively (Figure S2A). Quantitative reverse-transcription PCR (RT-qPCR) analyses revealed that all of these target genes were significantly upregulated in *ocn*-small interfering RNA (siRNA) (*ocn*-KD) cells, while except for *Ptp61F* and *Socs36E*, the others were all downregulated in *ocn* overexpressing cells (Figure 2B). This result indicates that Ocn negatively regulates JAK/STAT pathway. The upregulation of *Ptp61F* and *Socs36E* in cells overexpressing *ocn* might be attributed to their roles also as negative regulators of the JAK/STAT pathway.^4^

Given that Ocn is predicted to be a phosphatase, we then asked whether Ocn could dephosphorylate Stat and thus tend to inhibit its nuclear influx. Western blot analyses showed that the overexpression of Ocn in S2 cells (Figure 2F) led to a significant reduction in pSTAT relative to the control (Figure 2C). Correspondingly, *ocn*-KD in fly testes resulted in an increase in pSTAT (Figure 2D). These findings suggest that Ocn may function in directly or indirectly dephosphorylating Stat in flies.

Since Ocn may reduce the phosphorylation level of Stat, we proceeded to examine whether Ocn influences the translocation of Stat into the nucleus in S2 cells. In control cells, pSTAT signal was found in both nuclei and cytoplasm (Figure 2E). However, when cells were transfected with the construct carrying *ocn*, the pSTAT signal was significantly decreased in the nuclei. Instead, the cytoplasm exhibited strong pSTAT signal (Figure 2F). The nucleus/cytoplasm (N/C) ratio of the pSTAT signal per cell was significantly decreased in these cells relative to the control (0.27-fold, *p* < 0.001) (Figure 2H). Nevertheless, the N/C ratio of the pSTAT signal in *ocn*-KD cells was not significantly different from that in control cells (Figures 2G and 2I).

The pSTAT antibody used previously is against human phospho-STAT5A Y694, which corresponds to *Drosophila* Stat92E pY711, but Ocn is predicted to be a phosphohistidine phosphatase, we wondered if it might also have activity on phosphotyrosine. We therefore synthesized a pSTAT small peptide. This peptide represents the amino acid sequence surrounding Y711 of Stat92E (phosphorylated on this Y residue), which is conserved among STATs. Surprisingly, dot blot analysis revealed that Ocn could indeed dephosphorylate this pTyr peptide (Figures 2J and 2K). To confirm the specificity of dephosphorylation at Y711, we also synthesized a pSTAT small peptide, but with phosphorylation at S714. This peptide containing pS714 was then subjected to the same dot blot assay using pan pSer antibody. The result showed that Ocn failed to dephosphorylate the pS714 peptide (Figures S2B and S2C), in contrast to its clear activity toward the pY711 peptide.

Ocn contains a conserved site His-70, corresponding to mammalian PHPT1 His-53 and *Caenorhabditis elegans* (*C. elegans*) PHIP-1 His-45, which is required for phosphatase activity (Figure 6A).^23^ Mutation of the conserved H70 active site (H70A, with His-70 mutated to alanine) led to loss of Ocn’s ability to dephosphorylate Stat92E pY711 (Figures 2J and 2K). To further confirm that the dephosphorylation of the pY711 peptide is due to Ocn itself rather than a contaminating protein-tyrosine phosphatase (PTP), we added a general PTP inhibitor into the reaction solution that inhibits PTPs but not PHPT1 and found that Ocn-mediated dephosphorylation of Stat92E pY711 was reduced to 0.63-fold of the control (Figures 2L and 2M). The mass spectrometry (MS) assay also showed that the phosphate group on this peptide was present in the control solution without Ocn (Figure S2D) but was released when Ocn was added to the reaction solution (Figure S2E). These results demonstrate that Ocn is able to dephosphorylate STAT at this conserved tyrosine residue.

*Socs36E* is also the JAK/STAT pathway repressor. In GSCs, the expression of *Socs36E* fine-tunes JAK/STAT signaling to control their self-renewal.^4^ To further assay the interaction between *ocn* and JAK/STAT signaling from a genetic perspective, we generated flies through genetic hybridization with simultaneous KD of *ocn* and overexpressing *Socs36E* in early germline (*nosGal4* > *ocn-RNAi*; *UAS-Socs36E*) to determine whether overexpression of *Socs36E* could at least partially rescue the defects in male fertility induced by *ocn-*KD. Interestingly, we found that the overexpression of *Socs36E* indeed partially restored the male fertility, with the hatch rate of embryos fathered by the *nosGal4* > *ocn-RNAi*; *UAS-Socs36E* males increasing to 16.86% ± 7.40% (Table 1). Furthermore, immunofluorescence staining displayed that the testes from *nosGal4* > *ocn-RNAi*; *UAS-Socs36E* males contained some Vasa-positive cells, a few spermatid nuclear bundles in the base, and some mature sperm in the seminal vesicles (Figures 2N and 2O). These suggest that the phenotype of germ cell loss in adult flies following *ocn-*KD may be partially restored by overexpression of *Socs36E*. To further substantiate the role of Ocn in JAK/STAT signaling, we also knocked down *ocn* while concurrently overexpressing *Socs36E* (*ocn-KD*; *Socs36E-OE*) in S2 cells and then analyzed the expression of JAK/STAT target genes. RT-qPCR analyses confirmed the overexpression efficiency of *Socs36E* (Figure S2F). Our result showed that overexpression of *Socs36E* partially rescued the transcriptional aberrations caused by depletion of *ocn* (Figure S2G), which is consistent with male fertility phenotype observed *in vivo*. Taken together, these results indicate that Ocn negatively regulates the JAK/STAT signaling pathway through dephosphorylating *Drosophila* Stat92E on Y711.

**Table 1:** Overexpression of *Socs36E* partially rescues the infertility phenotype caused by *ocn* knockdown in germline in *D. melanogaster*

Cross group (♂ × ♀)	Egg hatch rate (%)	Egg counted	*p* value
(1) *nosGal4* > *w*^*-*^ × *w*^*1118*^	72.04 ± 4.06	1,359	−
(2) *nosGal4* > *UAS-socs36E* × *w*^*1118*^	22.12 ± 7.82	1,201	2 vs.1, *p* < 0.001
(3) *nosGal4* > *ocn-RNAi* × *w*^*1118*^	0	528	3 vs.1, *p* < 0.001
(4) *nosGal4* > *ocn-RNAi*; *UAS-socs36E* × *w*^*1118*^	16.86 ± 7.40	2,640	4 vs.1, *p* < 0.001

### *ocn*-KD in S2 cells leads to apoptosis

**Figure 3:**
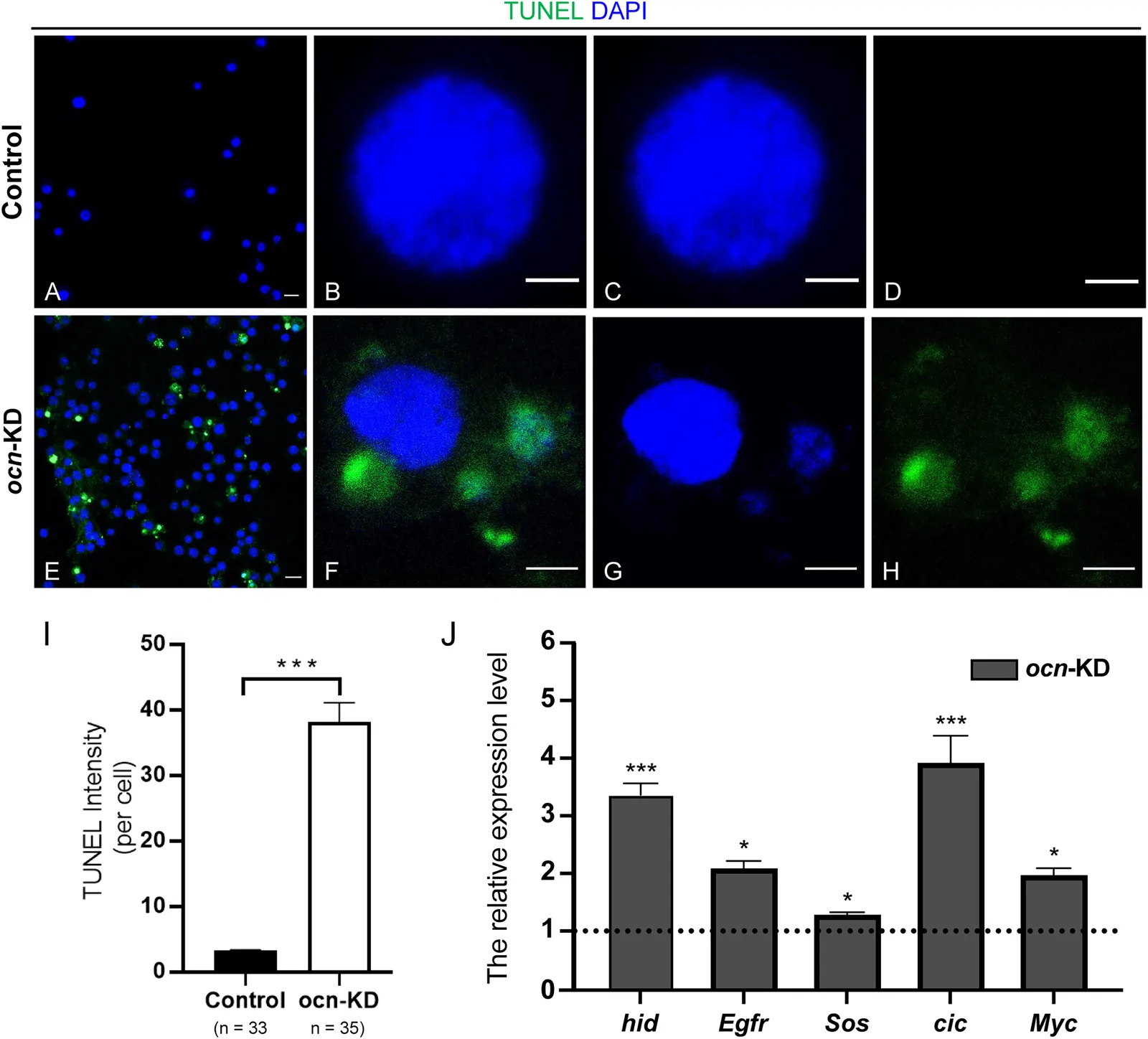
Knockdown of *ocn* in *Drosophila* S2 cells induced apoptosis (A–D) S2 cells transfected with a control construct. (E–H) S2 cells transfected with a construct containing *ocn*-siRNA (*ocn*-KD). Representative images are shown from two independent experiments, with 33 cells (control) and 35 cells (*ocn*-KD) analyzed. Scale bars: 10 μm in (A) and (E); 5 μm in (B)–(D) and (F)–(H). (I) The TUNEL fluorescence intensity in *ocn*-KD cells relative to the control cells. *n* represents the number of S2 cells. (J) Knockdown of *ocn* induced the upregulation of pro-apoptotic genes. Experiments were performed with at least three biological replicates. Data are shown as mean ± SEM. Student’s *t* test, ∗*p* < 0.05; ∗∗∗*p* < 0.001.

Curiously, we found that *ocn*-KD cells looked apoptotic. Therefore, we stained these cells with TUNEL and indeed observed much stronger TUNEL signals in cells expressing *ocn* siRNA (Figures 3E–3H) when compared to control cells (Figures 3A–3D). The TUNEL fluorescence intensity was significantly higher in *ocn-*KD cells than in the control cells, with an increase of approximately 8.86-fold (Figure 3I, *p* < 0.001).

Several pathways have been shown to link to Stat-involved apoptosis, including Hid, EGFR, and mitogen-activated protein kinase (MAPK),^24^^,^^25^^,^^26^ thus we detected the expression of certain members within these pathways, such as *hid*, *Egfr*, *Sos*, *cic*, and *Myc*. RT-qPCR analyses showed that all of these genes were significantly upregulated in *ocn*-KD cells (Figure 3J). To further demonstrate the involvement of Ocn in the JAK/STAT pathway, we examined these apoptosis-related gene expressions in S2 cells with *ocn*-KD and simultaneous *Socs36E* overexpression (*ocn-KD*; *Socs36E-OE*). This revealed that the transcription levels of all these genes were lower than those in the *ocn*-KD group (Figure S2H), indicating an inhibition of the upregulated expression of these apoptotic genes induced by *ocn*-KD. These results suggest that *ocn-*KD may trigger cell death and this process might be associated with the JAK/STAT pathway. This is in line with the observations in fly testes *in vivo*. Nevertheless, as we did not detect the pSTAT levels and nuclear localization, or apoptosis phenotypes, we acknowledge that the rescue data currently rest at the transcriptional and phenotypic level.

### *ocn*-KD induced the accumulation of Stat92E in the nuclei of somatic cells

**Figure 4:**
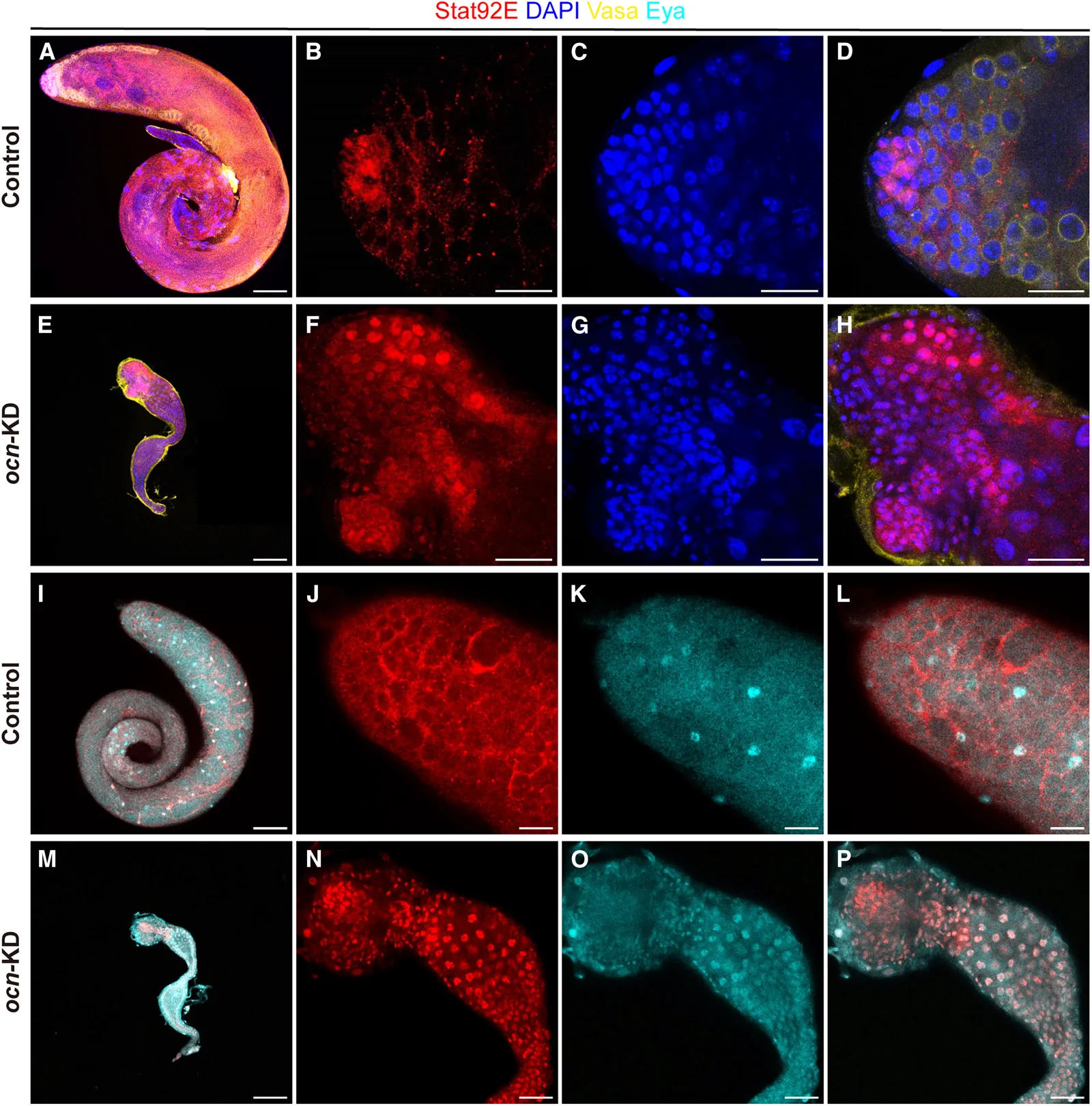
Stat92E accumulates in the nuclei of somatic cells in *ocn*-KD fly testes Fly testes were stained with anti-Stat92E antibody (red), Vasa antibody (yellow), Eya antibody (cyan), and DAPI (blue). (A–D and I–L) Control testes. (E–H and M–P) *ocn*-KD (*nosGal4*>*ocn-RNAi*) testes. (A, E, I, and M) The overall appearance of testes. (B–D, F–H, J–L, and N–P) Anterior regions of fly testes. Representative images from two independent experiments are shown. Scale bars, 100 μm (A, E, I, and M) and 25 μm (B–D, F–H, J–L, and N–P).

*ocn-*KD in S2 cells induced significant upregulation of *zfh1* and *chinmo*, which are main responders to JAK/STAT signaling in CySCs of fly testes. We therefore examined the expression of Stat92E protein and the development of somatic support cells in fly testes. As expected, Stat92E exhibited high expression mainly in GSCs that surrounded the hub at the tip of the control testis (Figures 4A, 4B, and 4D).^27^ Unexpectedly, although there were no germ cells in adult fly testes after *ocn-*KD (Figure 4H),^17^ Stat92E protein was expressed strongly not only at the apical region, but also in the subsequent anterior region (approximately the first half) of the small testes (Figures 4E, 4F, and 4H). Furthermore, the Stat92E was mainly enriched in the nuclei of the somatic cells in the small testes (Figures 4G and 4H).

Furthermore, the Stat92E signals were absent from the late stage cyst cells (labeled by Eya) in the control testes (Figures 4I–4L). However, in *ocn-*KD testes, Stat92E signals appeared clearly overlapping with Eya signals (Figures 4M–4P), indicating an accumulation of Stat92E in the nuclei of the cyst cells. These results suggest that the KD of *ocn* in early germ cells does not hinder the development of somatic cells. The increased Stat92E nuclear accumulation in somatic cells likely reflects a non-cell-autonomous activation of JAK/STAT signaling, potentially in response to germ cell loss in fly testes.

### *ocn* is required in somatic support cells for spermatogenesis

**Figure 5:**
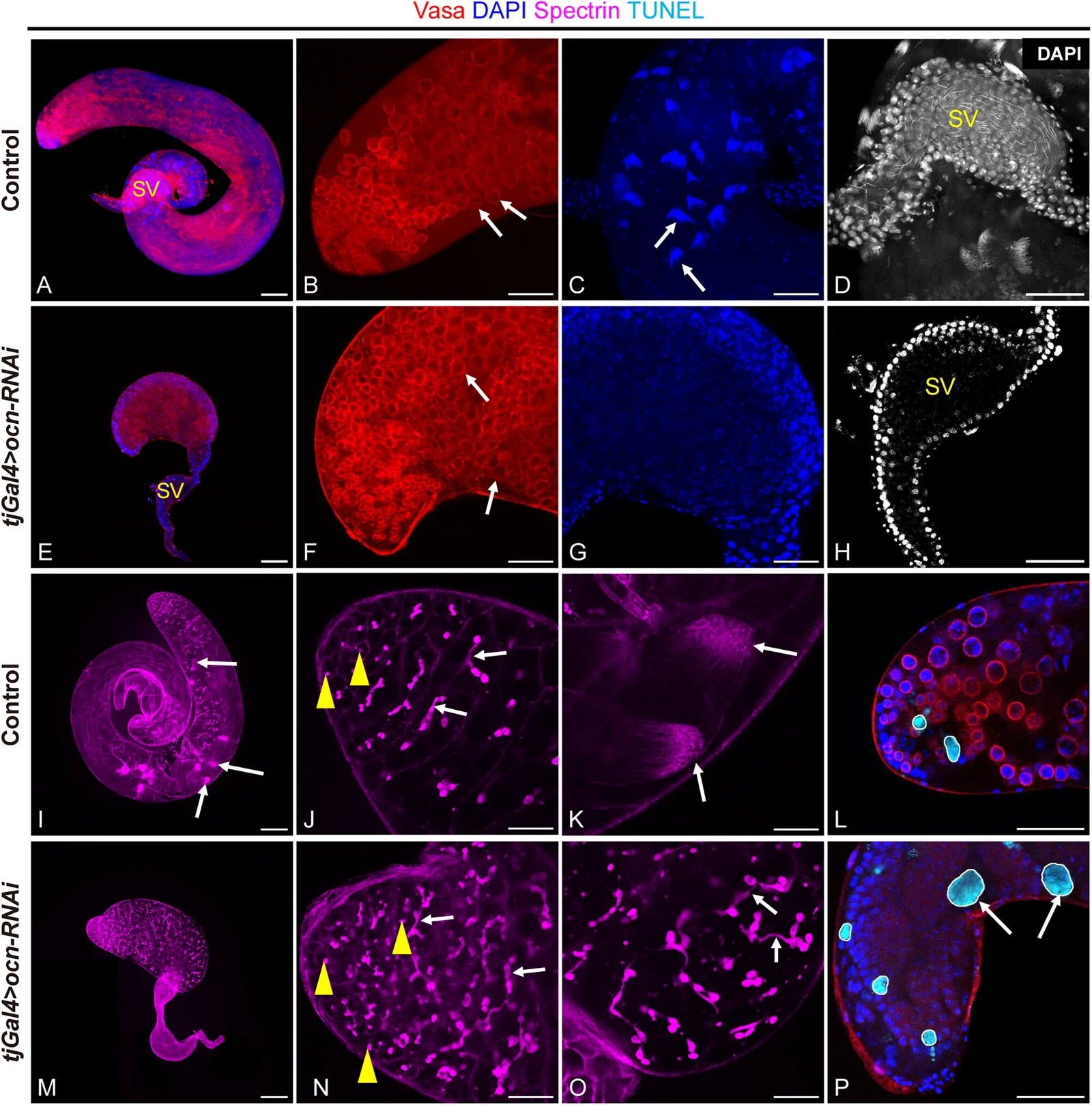
*ocn* is required in somatic support cells during spermatogenesis in *D. melanogaster* Anti-Vasa staining (red) labels germ cells, and DAPI (blue) marks nuclei. Anti-Spectrin (magenta) and TUNEL (cyan) mark fusomes and apoptotic cells, respectively. (A, E, I, and M) Overall appearance of the testes. (B, F, J, N, L, and P) Anterior portion of the testes. (C and G) Basal region of the testes. (D and H) Seminal vesicle (SV). (K and O) Middle region of the testes. Representative images from two independent experiments are shown. Scale bars, 100 μm (A, E, I, and M) and 25 μm (B–D, F–H, J–L, and N–P).

To study whether *ocn* is also required in somatic support cells for spermatogenesis and its relationship with JAK/STAT pathway, we knocked down *ocn* specifically in CySCs and early cyst cells using *tjGal4*. Approximately 76.94% (337/438) of *tjGal4* > *ocn-RNAi* fly testes were notably shorter and lacked a spiral tube shape (Figure 5E). Most regions of these testes exhibited swelling, with a sharp narrowing at the basal region (Figures 5A and 5E). Although Vasa-positive early germ cells and spermatocytes were present, indicating normal germ cell division and differentiation (Figure 5F), no differentiating spermatids were observed. In contrast to control testes, where spermatid nuclear bundles were visible in the basal region (arrows in Figure 5C), no such bundles were found in *tjGal4*>*ocn-RNAi* testes (Figure 5G). The seminal vesicles of *tjGal4*>*ocn-RNAi* testes were devoid of sperm (Figure 5H), while control seminal vesicles contained sperm with needle-like heads (Figure 5D).

To determine the developmental stage of the crowded germ cells distributed in the swollen region of the small *tjGal4*>*ocn-RNAi* testes, we stained the testes with spectrin antibody. In control testes, single GSCs and GBs exhibited round fusomes (arrowheads in Figure 5J), which became progressively more branched as the number of interconnected germline cells increased through mitotic divisions (Figures 5I and 5J). Spermatocytes displayed wider fusomes due to their larger cell size (arrows in Figure 5J). In *tjGal4*>*ocn-RNAi* testes, round fusomes appeared not only near the hub but also in areas farther away from the hub (arrowheads in Figure 5N). Furthermore, in the middle and basal regions of control testes many cylindrical spectrin-rich elongation cones were observed, indicating the growth ends of the sperm tails (arrows in Figures 5I and 5K). In contrast, even in the basal region, there were still wider branched fusomes (arrows in Figure 5O) characteristic of spermatocytes but lack any elongation cones in the small *tjGal4*>*ocn-RNAi* testes (Figure 5N). TUNEL staining revealed stronger signals in *tj*Gal4>*ocn-RNAi* testes, including in larger germ cell cysts (arrows in Figure 5P), compared to the control (Figure 5L). Together, these results suggest that *ocn* is also essential in somatic support cells for *Drosophila* spermatogenesis.

### Mammalian PHPT1 suppresses pSTAT nuclear localization in cells

**Figure 6:**
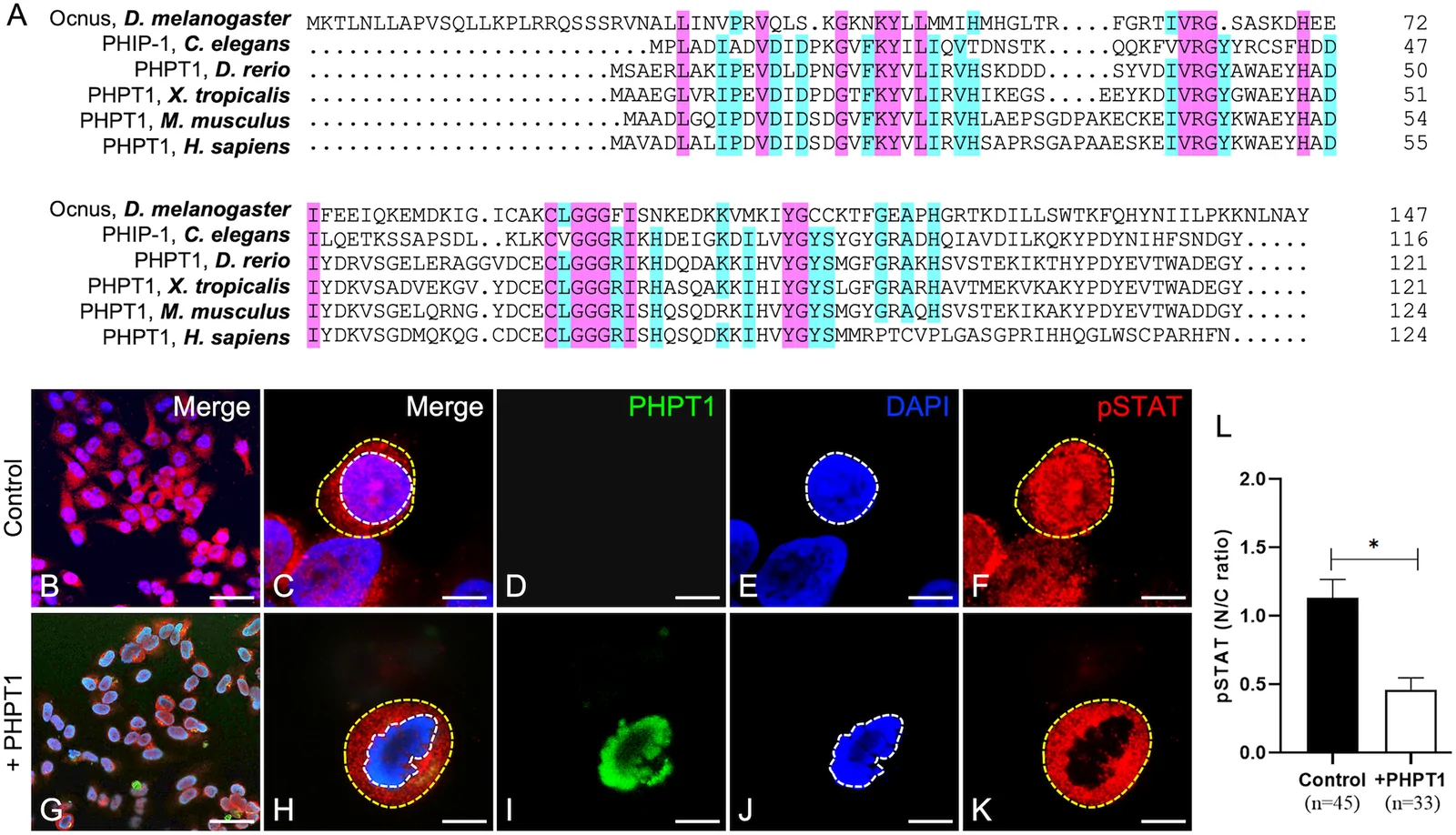
Expression of PHPT1 inhibits the nuclear localization of pSTAT in HeLa Cells (A) Multiple sequence alignment of *Drosophila* PHPT1 (Ocn) and its orthologs in model organisms. The amino acid sequences of *D. melanogaster*, *Caenorhabditis elegans* (*C. elegans*), *Danio rerio* (*D. rerio*), *Xenopus tropicalis* (*X. tropicalis*), *Mus musculus* (*M. musculus*), and *Homo sapiens* (*H. sapiens*) are from the NCBI database. The amino acid sequence of *Drosophila* PHPT1 is shown at the top. Purple indicates residues conserved in all six species, and blue indicates conserved residues in five species. (B–K) PHPT1 inhibits the nuclear localization of pSTAT. The subcellular localization of pSTAT5 in HeLa cells with the empty vector (B–F, Con) or with the vector carrying *PHPT1-GFP* fragment (G–K, PHPT1-OE). (C–F) Magnified views of cells in (B). (H–K) Magnified views of cells in (G). The white dashed circle outlines the nucleus, and the yellow dashed circle delineates the cell boundary. Representative images are shown from three independent experiments, with 45 cells (control) and 33 cells (PHPT1-OE) analyzed. Scale bars: 45 μm in (B) and (G); 8 μm in (C)–(F) and (H)–(K). (L) The nucleoplasmic fluorescence signal intensity of pSTAT is significantly reduced in PHPT1-expressing cells relative to control cells. *n* represents the number of cells from two independent repeat experiments. Data are represented as mean ± SEM. Student’s *t* test, ∗*p* < 0.05.

The human PHPT1 shares 49.4% sequence similarity with Ocn (Figure 6A). To investigate whether PHPT1 is associated with JAK/STAT signaling pathway, we expressed PHPT1 in HeLa cells. This induced an apparent decrease in nuclear localization of pSTAT (Figures 6G–6K) relative to the control cells without PHPT1 (Figures 6B–6F). Quantification of fluorescence intensity showed a significant reduction in the N/C ratio of pSTAT (0.46-fold, *p* < 0.05) compared to control cells (Figure 6L). These findings suggest that PHPT1 may inhibit the nuclear translocation of pSTAT, potentially serving as a target for reducing cancer progression.

Since our initial research focused on *Drosophila* male germ cells, we further expressed mouse PHPT1 in mouse spermatogenic GC-2 cells. As the previous study have shown that *ocn*-KD in cells may lead to apoptosis (Figures 3E–3H), to minimize the impact of apoptosis on the experimental results as much as possible, we performed immunofluorescence staining 24 h after transfection. The results exhibited that PHPT1 expression in GC-2 cells (+PHPT1) induced an evident decrease in nuclear localization of pSTAT (Figures S3F–S3J) relative to the control cells without PHPT1 (Figures S3A–S3E). Quantification of fluorescence intensity showed a significant reduction in the N/C ratio of pSTAT (0.07-fold, *p* < 0.001) compared to control cells (Figure S3P). The KD group (PHPT1-KD) showed a significantly increased N/C ratio of pSTAT relative to the +PHPT1 group (*p* < 0.001), although this ratio remained lower than that in the control group (Figures S3K–S3P). This may be associated with the initiation of apoptosis in these cells.

## Discussion

Our previous work has demonstrated that *Drosophila PHPT1* (*ocn*) plays a crucial role in male germ cell development.^17^ In this study, we extend these findings, elucidating the role of *ocn* in regulating germ cell survival during larval stages and its involvement in JAK/STAT signaling. We demonstrate that as a negative regulator, *ocn* depletion stimulates activation of JAK/STAT signaling by dephosphorylating the conserved Y711 site of Stat92E, influencing the nuclear localization of pSTAT and its downstream gene expressions, leading to apoptosis. Importantly, we showed that human PHPT1 exerts a similar effect on pSTAT nuclear dynamics in HeLa cells. This suggests an evolutionarily conserved mechanism of nuclear accumulation of pSTAT among animals.

Our prior research has shown that *ocn*-KD in the early germline results in germ cell loss in adult male testes.^17^ However, it remains unclear when this germ cell loss occurs during development. In *Drosophila*, approximately five nuclei reach the surface of the embryo’s posterior pole during the ninth nuclear division cycle. These nuclei are enclosed by cell membranes and form the PGCs. During gastrulation, around 30 PGCs are internalized along with the endoderm and then migrate through the epithelium, and eventually coalesce with the somatic gonad by embryonic stage 14.^28^ Maternally synthesized *nanos* (*nos*) RNA has been shown to play a crucial role in the germ cell specification and migration.^29^ Given that we used a *nosGal4* driver to KD *ocn*, we questioned whether this could affect the specification and migration of PGCs. We here observed that *ocn*-KD did not impair the specification and migration of PGCs. The PGCs could normally produce and migrate to form the embryonic gonad, suggesting that *ocn* is not involved in the formation and migration of PGCs in male *D. melanogaster*.

During the embryo to larval transition, PGCs in the developing male gonad differentiate into GSCs upon the establishment of the embryonic hub. In the 1^st^ instar larval testes, the GBs commences mitotic division. By the 2^nd^ instar larval stage, the germ cell cysts containing 2–16 germ cells are present in the testes.^30^ Although the testicular sizes of the 1^st^ instar *ocn*-KD larvae are almost identical to those of the control group, a notable difference is seen in the 2^nd^ instar *ocn*-KD larvae. These larvae exhibit much fewer germ cells and almost no cysts containing more than two germ cells. Especially, by the 3^rd^ instar larval stage, the *ocn*-KD testes are markedly smaller than the controls, with no germ cells identified by Vasa staining. Conversely, the Fas III signal indicative of hub cells is evidently extended, which could be attributed to germ cells loss.^31^ These suggest that the loss of germ cells caused by *ocn*-KD occurs mainly during the 2^nd^ instar larval stage when the spermatogonia undergo transit-amplification to produce spermatogonial cysts.^13^^,^^30^ Therefore, *ocn*-KD disrupts the mitotic divisions during transit-amplification of spermatogonia.

Upon the formation of the hub, the stem cell niche in *Drosophila* testes, is established, it activates the critical JAK/STAT signaling pathway, which regulates the development of the cells that associate with the hub. Cells adjacent to the hub express stem cell markers, including Stat92E, while those not associated with the hub begin to differentiate.^18^^,^^30^ The small testes observed in the 3^rd^ instar larvae of *ocn*-KD flies are reminiscent of the phenotype induced by the defects in JAK/STAT pathway.^18^ Using *Drosophila* S2 cells, we here found that *ocn* negatively regulated the expression of Stat92E target genes. Furthermore, a significantly decreased N/C ratio of pSTAT was observed in *ocn* overexpressing cells, which may account for the downregulation of Stat92E target genes. Especially, we demonstrate by both dot blot and MS assays, that chemically pSTAT peptide containing the well-conserved tyrosine (Y711 of *Drosophila* Stat92E) is also a substrate of Ocn. To our knowledge, this substrate relationship has not been previously described.

Previous studies reported that mammalian PHPT1 did not exhibit pTyr peptide dephosphorylation,^32^^,^^33^ although we were unable to find corresponding figures or other forms of result presentation except for textual descriptions. However, our combination of dot blot, mutation of the key catalytic residue, PTP inhibition assays, and MS analysis collectively demonstrated that Ocn has certain tyrosine phosphatase activity. This discrepancy may be due to differences in detection methods, as their studies used microplate readers and autoradiography. While these methods are conventional, they are inherently limited by relatively low sensitivity. Microplate reader assays require accumulation of detectable amounts of free phosphate and thus are prone to false negative results when the enzyme exhibits weak or modest activity toward a given substrate. Autoradiography, although more sensitive, suffers from high background noise and requires careful optimization to avoid signal masking. These limitations are particularly problematic when the tyrosine phosphatase activity of PHPT1/Ocn is not very strong. In contrast, our study employed dot blot (including mutation of the key catalytic residue, PTP inhibition) using the specific and conserved anti-pSTAT antibody, coupled with a high-sensitivity, MS-based assay. This combination allowed us to functionally confirm the activity of PHPT1/Ocn and directly and sensitively identify substrate sequences and phosphorylation sites. Therefore, we propose that the certain tyrosine phosphatase activity of PHPT1/Ocn has been previously overlooked, largely due to methodological constraints. Our results indicate that, apart from pHis and pLys,^34^ *Drosophila* PHPT1/Ocn also acts on phosphotyrosine. This finding reveals a broader specificity for this enzyme than known so far and thus may have much wider functions in animal development.

JAK/STAT signaling plays a pivotal role in development, including spermatogenesis.^4^^,^^5^^,^^35^ However, interpreting the JAK/STAT pathway during development is challenging due to its multifaceted and intricate roles. For example, genetic studies have previously shown that activation of *Drosophila* JAK/STAT pathway results in dramatically overgrown adult eyes, while downregulation of this pathway leads to small or absent eyes.^36^^,^^37^ Conversely, other research has reported that activation of the JAK/STAT signaling induces *hid*-mediated apoptosis during eye development and oogenesis.^24^^,^^38^^,^^39^ A study on larval wing disc development by Mukherjee et al. also revealed that *Drosophila* Stat92E possesses both the pro-proliferative and the anti-proliferative functions.^40^ These findings collectively suggest that the JAK/STAT pathway can induce both proliferation and apoptosis during development in a context-dependent manner in *Drosophila*. In this study, we observed that *ocn*-KD led to JAK/STAT hyperactivation and enhanced apoptosis, both *in vivo* and *in vitro*. Furthermore, *ocn*-KD in S2 cells leads to significant upregulation of several apoptosis-related members, including *hid*, *EGFR*, and MAPK pathway. It is possible that JAK/STAT hyperactivation induced by *ocn*-KD directly promotes apoptosis in this context. Alternatively, the increased pSTAT levels and apoptosis may be independent consequences of *ocn*-KD, both driven by a common upstream signal. Given the complexity of cell types in *Drosophila* testis, studies on expression changes of both Stat92E target genes and apoptosis-related genes were performed in S2 cells. Nevertheless, further *in vivo* or *in situ* validation of gene expression is required to better elucidate the underlying mechanisms.

It is worth noting that, in addition to the canonical role of tyrosine-pSTAT in transcriptional activation, several studies have reported that unphosphorylated STAT (uSTAT) proteins can also localize to the nucleus and associate with heterochromatin protein 1 (HP1) to maintain heterochromatin stability.^41^^,^^42^ Thus, Ocn-mediated dephosphorylation may serve a dual role: inhibiting the deleterious effects of excessive pSTAT signaling while concurrently generating the uSTAT pool competent for heterochromatin-associated functions. Hence, apoptosis observed after *ocn*-KD could primarily be attributable to pSTAT-driven hyperactivation; however, the concomitant depletion of uSTAT and potential compromise of heterochromatin integrity may also be a contributing factor. Therefore, whether Ocn also regulates the nuclear distribution or chromatin-associated functions of unphosphorylated Stat92E warrants further investigation.

JAK/STAT signaling is a crucial signaling pathway in human cellular functions, including cell proliferation, differentiation, survival, and apoptosis, and its dysregulation has been linked to various pathological defects, including cancer.^43^^,^^44^ Our research indicates that the human ortholog of Ocn, PHPT1, also negatively regulate this pathway. Ectopic expression of PHPT1 in HeLa cells significantly reduced the N/C ratio of pSTAT, suggesting a decrease in JAK/STAT activity. PHPT1 has been shown to regulate cell proliferation, differentiation, and regeneration.^23^^,^^34^^,^^45^ Notably, most studies seem to show that PHPT1 acts as a promoter of different cancer progressions, such as hepatocellular carcinoma, lung, and pancreatic cancers, and clear cell renal carcinoma.^26^^,^^34^^,^^46^^,^^47^ For instance, PHPT1 is highly expressed in hepatocellular carcinoma tissues and cell lines, and its KD inhibits cell proliferation and promotes cell apoptosis.^46^ Additionally, PHPT1 expression levels are also elevated in EGFR mutant lung cancer cells. Inhibition of PHPT1 expression in these cells significantly suppressed their proliferation.^26^ Our findings that *ocn*-KD induces cell apoptosis in *Drosophila*, both *in vivo* and *in vitro* are consistent with these human cell studies. This suggests that PHPT1/Ocn may function as a conserved negative regulator of the JAK/STAT signaling pathway across invertebrates and vertebrates, and that its downregulation leads to JAK/STAT hyperactivation and apoptosis. Given that JAK/STAT dysregulation is implicated in a wide spectrum of human diseases, including cancers and immune disorders,^48^^,^^49^ and that pro- or anti-tumorigenic roles of JAK/STAT signaling are highly context-dependent,^50^ our findings may provide previously unrecognized perspectives for developing cancer diagnostics and targeted therapeutic interventions. For example, in cancers with aberrant overexpression of PHPT1, such as EGFR-mutant lung cancer where PHPT1 promotes cell proliferation and drug resistance,^26^ elevated levels of PHPT1 may lead to hypoactivity of JAK/STAT signaling, which could contribute to carcinogenesis likely through cross talk with immune pathways and/or other oncogenic pathways. Therefore, developing specific PHPT1 inhibitors through high-throughput screening or structure-based drug design using the available crystal structure, would be predicted to induce apoptosis and thereby suppress carcinoma cell proliferation, which may be mediated by activating the JAK/STAT signaling pathway to trigger immune responses. Conversely, in tumors characterized by hyperactivation of the JAK/STAT signaling, enhancing PHPT1 activity may inhibit excessive activation of the JAK/STAT pathway, which would be expected to reduce the expression of its downstream pro-tumorigenic targets and thus inhibit cancer cell proliferation. Unlike many classical PTPs that are large, redox sensitive, and often lack substrate specificity, PHPT1/Ocn is a small (∼14 kDa) enzyme that appears to have relatively restricted substrate preference. Its small size and simple structure may facilitate the development of small molecule modulators. Thus, PHPT1 activators could potentially counteract pathological STAT hyperactivation, whereas PHPT1 inhibitors might be beneficial in settings where STAT activity is suboptimal. The anti-tumor efficacy of these agents should be validated in preclinical models, such as cancer cell lines and genetically engineered mouse models. Combination strategies with existing JAK inhibitors could also be explored to achieve synergistic effects. Moreover, the expression level or enzymatic activity of PHPT1/Ocn could be explored as a potential diagnostic or prognostic biomarker in diseases associated with aberrant STAT signaling. Future studies using genetic and pharmacological approaches in relevant disease models are warranted to explore these possibilities.

In conclusion, this study identifies a previously unrecognized pathway involved in *Drosophila* spermatogenesis. *Drosophila* PHPT1/Ocn may participate in spermatogenesis by negatively regulating the JAK/STAT pathway and control apoptosis during mitotic division. Notably, this mechanism appears to be conserved, as evidenced by the similar regulation of N/C ratio of pSTAT by PHPT1 within human cells. Given that JAK/STAT signaling is involved in the initiation and progression of many diseases, including tumorigenesis, our findings may extend current understanding of the potential for developing cancer diagnostic and therapeutic strategies.

### Limitations of the study

Although we identified Stat92E as a direct substrate of PHPT/Ocn and confirmed the ability of Ocn to dephosphorylate the conserved site Y711 by dot blot and MS analysis, *in vivo* studies using transgenic or mutant *Drosophila* that validate the requirement for this catalytic activity during spermatogenesis are still lacking. Furthermore, while we demonstrated that Ocn is essential for *Drosophila* spermatogenesis and human PHPT1 similarly suppressed pSTAT nuclear accumulation in HeLa cells, whether PHPT1 is also required for spermatogenesis in mammals by regulating JAK/STAT signaling is not experimentally investigated in this study. Future studies in *Drosophila* could focus on generating transgenic lines expressing a catalytically inactive mutant of *ocn* to determine whether its phosphatase activity is essential for spermatogenesis. Rescuing the *ocn* KD phenotype with wild-type versus catalytically dead transgenes would provide direct genetic evidence that the observed defects are specifically attributable to Ocn’s phosphatase activity. In parallel, testing whether human *PHPT1* can rescue the *ocn* loss-of-function phenotype in *Drosophila* would provide functional evidence for evolutionary conservation. Generating germ cell-specific or whole-body *PHPT1* knockout mammalian models would allow investigation of whether PHPT1 plays a role in mammalian spermatogenesis through JAK/STAT regulation. Such studies will not only validate the physiological relevance of our findings but also explore their translational potential for treating JAK/STAT-driven diseases.

## Resource availability

### Lead contact

Requests for further information and resources should be directed to and will be fulfilled by the lead contact, Yu-Feng Wang (yfengw@ccnu.edu.cn).

### Materials availability

This study did not generate new unique reagents. All fly stocks, plasmids, antibodies, and reagents used in this study are commercially available or available from the sources listed in the key resources table.

### Data and code availability

• This study did not generate or analyze any large-scale datasets.
• This paper does not report original code.
• Any additional information required to reanalyze the data reported in this paper is available from the lead contact upon reasonable request.
• Unedited microscopy images from at least two independent experiments have been deposited in Figshare and are publicly available at https://doi.org/10.6084/m9.figshare.33173567.

## Acknowledgments

We thank Professor Zhaohui Wang at the Institute of Genetics and Developmental Biology, Chinese Academy of Sciences for anti-Stat92E antibody, Xian-Wei Wang at Shandong University for anti-pSTAT5 antibody, Xi Zhou at the Wuhan Institute of Virology, Chinese Academy of Sciences for S2 cells, and Guang Song at Central China Normal University for HeLa cells. This work was supported by the 10.13039/501100001809National Natural Science Foundation of China (32170511), the Knowledge Innovation Program of Wuhan-Basic Research (2023020201010122), the 10.13039/501100012226Fundamental Research Funds for the Central Universities (CCNU25JC042), and the project of Key Laboratory of Pesticide & Chemical Biology, 10.13039/501100002338Ministry of Education of China (KL202601).

## Author contributions

Conceptualization, Y.-F.W., K.L., and J.-J.T.; methodology, Y.-F.W., K.L., J.-J.T., Q.W., Y.R., and B.M.; investigation, Q.W., Y.R., and M.-Y.C.; writing – original draft, Q.W., Y.R., Y.-F.W., and X.G.; writing – review and editing, Q.W. and Y.-F.W.; funding acquisition, Y.-F.W.; resources, X.G. and Y.-F.W.; supervision, Y.-F.W., K.L., and J.-J.T.

## Declaration of interests

The authors declare that they have no conflict of interest.

## STAR★Methods

### Key resources table

**Table:** 

REAGENT or RESOURCE	SOURCE	IDENTIFIER
**Antibodies**		

Rat anti-Vasa	Developmental Studies Hybridoma Bank (DSHB)	Cat# anti-vasa; RRID: AB_760351
Mouse anti-Fasciclin III	DSHB	Cat# 7G10 anti-Fasciclin III; RRID: AB_528238
Mouse anti-Eya	DSHB	Cat# eya10h6; RRID: AB_528232
Mouse anti-Armadillo	DSHB	Cat# N2 7A1 ARMADILLO; RRID: AB_528089
Mouse anti-Spectrin	DSHB	Cat# 3A9; RRID: AB_528473
Rabbit anti-Stat92E	Gift from Zhaohui Wang (IGDB, CAS)^51^	https://doi.org/10.1016/j.ydbio.2013.01.023
STAT5 Rabbit mAb	ABclonal	Cat# A5029; RRID: AB_2863420
Phospho-STAT5-Y694 Rabbit pAb	ABclonal	Cat# AP0887; RRID: AB_2771572
Rabbit anti-pan phosphoserine antibody	ABclonal	Cat# AP1475; RRID: AB_3698468
Beta Actin Polyclonal antibody	Proteintech	Cat# 20536-1-AP; RRID: AB_10700003
mCherry Polyclonal antibody	Proteintech	Cat# 26765-1-AP; RRID: AB_2876881
GFP tag Polyclonal antibody	Proteintech	Cat# 50430-2-AP; RRID: AB_11042881
Anti-GFP-Tag Rabbit mAb conjugated Magnetic Beads	Engibody	Cat# AT0041
Anti-mCherry tag Mouse mAb conjugated Magnetic Beads	Engibody	Cat# AT2104
HRP-conjugated Rabbit Anti-Goat IgG(H + L)	Proteintech	Cat# SA00001-4; RRID: AB_2864335
Goat Anti-Rat IgG(H + L) (Alexa Fluor® 488 Conjugate) Antibody	Absin	Cat# abs20196
Goat anti-Rabbit IgG(H + L) (AF594 Conjugate) Antibody	Absin	Cat# abs20164
Goat Anti-Rat IgG(H + L) (Alexa Fluor® 647 Conjugate) Antibody	Absin	Cat# abs20197
CoraLite594-conjugated Goat Anti-Mouse IgG(H + L)	Proteintech	Cat# SA00013-3; RRID: AB_2797133

**Biological samples**		

*Drosophila melanogaster*	This study	N/A

**Chemicals, peptides, and recombinant proteins**		

Schneider’s Drosophila medium	Gibco	Cat# 11720-034
DMEM	Gibco	Cat# 11965-092
Fetal bovine serum	Gibco	N/
Lipofectamine 2000	Invitrogen	Cat# 11668019
FuGENE HD	Promega	Cat# E2311
PTP inhibitor 1	MedChemExpress	Cat# HY-W013478
Phosphorylated Stat92E peptide (Y711)	Shanghai Apeptide	Custom
Mounting Medium, Antifading (with DAPI)	Solarbio	Cat# S21133

**Critical commercial assays**		

*In situ* Cell Death Detection Kit, Fluorescein	Roche	Cat# 11684795910

**Deposited data**		

Raw microscopy images	This study	https://doi.org/10.6084/m9.figshare.33173567

**Experimental models: Cell lines**		

*Drosophila* S2 cells		N/A
HeLa cells	Gift from Guang Song	N/A
mouse spermatogenic GC-2 cell line	Procell system	Cat# CL-0593

**Experimental models: Organisms/strains**		

nos-Gal4	Bloomington Drosophila Stock Center	BDSC#4937
*ocn*-RNAi	Tsinghua Fly Center	THU1774
UAS-*Socs36E*	FlyORF	F0015914
tj-Gal4/CyO	Kyoto Drosophila Stock Center (formerly DGRC)	DGRC#104055

**Oligonucleotides**		

ocn siRNA	Wuhan Qijing Biotechnology	See Table S1
qPCR primers	This study	See Table S1

**Recombinant DNA**		

pAc5.1-EGFP-Ocn	This study	N/A
pAc5.1-EGFP-OcnH70A	This study	N/A
pAc5.1-mCherry-Stat92E	This study	N/A
pAc5.1-Scos36E	This study	N/A
pcDNA3.1(+)-N-eGFP-mPHPT1(mouse PHPT1)	This study	N/A
pcDNA3.1(+)-N-eGFP-hPHPT1(human PHPT1)	This study	N/A

**Software and algorithms**		

ImageJ/FIJI	NIH	https://imagej.nih.gov/ij
R software	R Foundation	https://www.r-project.org
ClustalW	EMBL-EBI	https://www.clustal.org
DNAMAN	Lynnon Biosoft	N/A
LAS X	Leica Microsystems	N/A

### Experimental model and study participant details

#### Drosophila melanogaster

The *nosGal4* (BDSC 4937) was sourced from the Bloomington Drosophila Stock Center (USA). The *ocn*-RNAi line (THU1774) was obtained from the TsingHua Fly Center (Beijing, China). The *UAS-Socs36E* line (F0015914) was obtained from FlyORF (Zurich, Switzerland). The *tjGal4/Cyo* line (DGRC 104055) was acquired from the Kyoto Drosophila Stock Center (formerly DGRC) (Kyoto, Japan). The flies were kept on a standard cornmeal/yeast diet at 25°C with a 12/12 light/dark cycle.^19^ Developmental stages and sexes are specified in the corresponding figure legends.

#### Cell lines

*Drosophila* S2 cells were cultured at 27°C in *Drosophila* Schneider's medium supplemented with 10% heat-inactivated fetal bovine serum and 1% penicillin-streptomycin.

HeLa cells, a kind gift from Professor Guang Song and originally obtained from ATCC, and the mouse spermatocyte cell line GC-2spd(ts) from Procell, were cultured in DMEM supplemented with 10% FBS and 1% penicillin-streptomycin at 37°C in a 5% CO_2_ incubator. Cells were routinely tested negative for mycoplasma using the GMyc-PCR Mycoplasma Test Kit (Yeasen).

### Method details

#### Cell culture and transfection

Cell culture and transfection were conducted as previously reported.^52^ For S2 cells, plasmids or siRNAs were transfected using Lipofectamine 2000 according to the manufacturer’s instructions. Cells were harvested 48 h post-transfection for subsequent analyses, unless otherwise specified.

Cells were plated in 12-well plates and transfected with 4 μg of either plasmid or an empty vector using FuGENE HD.

#### Site-directed mutagenesis

Site-directed mutagenesis was performed using the Fast Mutagenesis System (FM111, TransGen Biotech) according to the manufacturer’s instructions. Based on the pAc5.1-ocn plasmid, the histidine at position 70 was substituted with alanine (OcnH70A). Primers used for mutagenesis are listed in Table S1. The mutation was verified using DNA sequencing. The *ocn* cDNA or *ocnH70A* was cloned into the pAc5.1-EGFP plasmid to create pAc5.1-EGFP-ocn or pAc5.1-EGFP-ocnH70A, respectively, while *stat92E* cDNA was cloned into pAc5.1-mCherry to produce pAc5.1-mCherry-Stat92E.

#### Immunofluorescence

Immunostaining was performed as described previously.^14^ Embryos were dechorionated in bleach, rinsed with water, and fixed in a heptane and 4% paraformaldehyde (PFA) mixture (1:1) for 30 min, followed by vigorous shaking with methanol before storage in methanol.^53^ Larvae and adult testes were dissected in PBS, pH 7.4, and fixed with 4% PFA for 30 min at room temperature (RT). Transfected cells (1×10^6^ per well) were fixed with 4% PFA after 48 h of growth on coverslips. All samples underwent three washes with PBS-T, blocking with 5% goat serum, and incubation with primary antibodies for 3-5 h at RT or overnight at 4°C. After incubation with secondary antibodies and DAPI, samples were mounted. The working concentration and source of primary antibodies were used as follows: rat anti-Vasa (1:200, Developmental Studies Hybridoma Bank, DSHB, AB_760351), mouse anti-Fasciclin III (1:100, DSHB, AB_528238), mouse anti-Eya (1:100, DSHB, AB_528232), mouse anti-Armadillo (1:200, DSHB, AB_528089), mouse anti-Spectrin (1:600, DSHB, AB_528473), rabbit anti-Stat92E (1:5000. Due to its limited quantity, it was solely used to stain fly testes. For Western blot, we used anti-STAT5) from Professor Zhaohui Wang at the Institute of Genetics and Developmental Biology, Chinese Academy of Sciences,^51^ rabbit anti-STAT5 (1:200, ABclonal, A5029), rabbit anti-pSTAT5 (1:200, ABclonal, AP0887). Secondary antibodies conjugated to Alexa Fluor 488, 594, and 647 (Absin, abs20196, abs20164, and abs20197) were diluted at 1:500. Confocal images were captured with a confocal microscope DMI8 Leica SP8 driven by LasX software, using 63x oil or 20x immersion lenses. The size of the third instar larval testis and the relative fluorescent intensity were measured using ImageJ/FIJI and normalized by mean.

#### Co-Immunoprecipitation and western blot

*Drosophila* S2 cells were transfected with pAc5.1-EGFP-Ocn and pAc5.1-mCherry-Stat92E plasmids, and after 48 h, the cells were lysed in ice-cold buffer and centrifuged to collect the supernatant. A fraction of the supernatant was incubated overnight with anti-mCherry immunomagnetic beads, followed by washing, resuspension in SDS buffer, and boiling to elute the proteins. The samples were separated by SDS-PAGE, transferred to nitrocellulose membranes, blocked with 5% milk in TBS-T, and probed with specific primary antibodies, including rabbit anti-mCherry (1:2500, Proteintech, 26765-1-AP), rabbit anti-GFP (1:2500, Proteintech, 50430-2-AP), rabbit anti-beta-Actin (1:3000, Proteintech, 20536-1-AP), rabbit anti-STAT5, and rabbit anti-pSTAT5. After secondary antibody incubation, the membranes were washed, visualized using ECL, and imaged to detect protein signals. The pSTAT levels were normalized to Actin, which served as the loading control. As the total STAT bands were less well resolved than the Actin bands, the Actin signal provided a more robust and consistent reference for normalization; at identical levels of actin, total STAT levels were nearly comparable.

#### RT-qPCR

Total RNA was extracted using Trizol according to the manufacturer's recommendations. First-strand cDNA was then synthesized from 2 μg of total RNA using the All-in-Mix 1st Strand cDNA Synthesis Kit. qPCR was performed on a BIO-RAD Miniopticon system using SYBR Green SupMix. The qPCR cycling program was 95°C for 3 min, followed by 40 cycles of 95°C for 10 s, 60°C for 30 s, and 72°C for 20 s. The relative expression of the genes was calibrated against the reference gene *rp49* using the 2^–ΔΔCt^ method^52^: ΔΔCt = (Ct_Target_− Ct_rp49_) Tested sample − (Ct_Target_− Ct_rp49_) Corresponding control. All experiments were conducted with at least three biological replicates. The primers were designed based on sequences in the Flybase (flybase.org) and shown in Table S1.

#### *In vitro* phosphatase assays

A synthetic peptide corresponding to amino acids 707–715 of Stat92E (PVTGYVKST), with phosphorylation at Y711 (which is well conserved among STATs), was synthesized by Shanghai Apeptide Co.,Ltd. (China). S2 cells were seeded into 12-well plates. 12 h later, cells were transfected with pAc5.1-EGFP-ocn or pAc5.1-EGFP-ocnH70A plasmids (1 μg/well). After 48 h, cells were lysed and incubated overnight at 4 °C with eGFP antibody-conjugated magnetic beads for purification. The synthetic phosphorylated peptide (10 mM) was incubated separately with equal concentrations of purified Ocn or OcnH70A in a reaction buffer containing HEPES (50 mM, pH 7.4) and MgCl_2_ (5 mM) at 30°C for 1 hour at a protein-to-peptide volume ratio of 1:1 (total reaction volume = 2 μL).^23^ For the control group, PBS was added instead of protein to ensure equal reaction volumes. To determine whether Ocn possesses tyrosine phosphatase activity, the reaction solution included 10 μM PTP inhibitor 1.

For the dot blot assay, equal volumes of samples were spotted onto a nitrocellulose membrane and blocked with rapid blocking buffer. The membrane was incubated overnight at 4°C with anti-pSTAT antibody (1:3000) or pan-phosphoserine antibody (1:2000, ABclonal, AP1475), followed by three washes with TBST (5-10 min each). Subsequently, it was probed with HRP-conjugated antibody (1:5000, Proteintech) for 1 h at room temperature. After three additional TBST washes (5-10 min each), the membrane was visualized using a chemiluminescence imaging system. Dot signal density was measured using ImageJ software and normalized to the control group (*n* = 4).

For MS assay, after incubation with Ocn, the peptide samples were desalted and resuspended in 0.1% formic acid in water and analyzed using an Orbitrap Exploris 480 high-resolution mass spectrometer coupled with a Vanquish Neo UHPLC system. Peptides were separated on a homemade C18 column (15 cm × 75 μm, 3 μm particle size, 100 Å pore size) at a flow rate of 0.3 μL/min. Peptide elution was performed using a gradient from 5% to 80% mobile phase B (mobile phase A: 0.1% formic acid water; mobile phase B: 0.1% formic acid ACN). MS analysis was conducted in data-dependent acquisition (DDA) mode with the following parameters: full MS scans at a resolution of 70,000, m/z range 350–2,0000, automatic gain control (AGC) target of 3 × 106, and maximum injection time of 20 ms; MS/MS scans with an AGC target of 5 × 104, maximum ion time of 50 ms, minimum signal threshold of 4 × 103, dynamic exclusion time setting 40 s, and exclusion of unassigned and singly charged ions.

#### TUNEL assay

The TUNEL assay was conducted using the ApopTag *in situ* apoptosis detection kit (Roche). Samples were dissected, fixed, washed, and incubated with a TUNEL reaction mixture (5 μl TdT enzyme solution and 45 μl fluorescein-dUTP tag solution) at 37°C in the dark for 3 h. After washing three times with PBS-T (10 min each), the samples were mounted on glass slides using antifade medium containing 2 μg/ml DAPI. A Leica SP8 laser confocal microscope was used to observe and take photos. All images were acquired using identical confocal microscope settings for the control and experimental groups. Germ cell types were identified by examining Z-stack images based on anti-Vasa staining. Within the identified germ cells, DAPI staining was used to confirm nuclear localization, and ROIs were manually selected as DAPI-positive nuclei showing clear TUNEL-positive signals. Fluorescence intensity was quantified using the Measure function in ImageJ (NIH), and mean fluorescence intensity was defined as the mean gray value within each ROI. The average fluorescence intensity of TUNEL-positive nuclei in a single testis was calculated, with each data point representing one testis. For S2 cells, since nuclei without TUNEL signals remained intact with DAPI staining comparable to that of the control, whereas all nuclei exhibiting TUNEL signals showed fragmentation, it was assumed that cells lacking TUNEL signals were those not transfected with *ocn*-siRNA. Thus, the measured TUNEL signal intensity per cell specifically reflected values obtained from TUNEL-positive cells.

#### Bioinformatics analysis

The Ocn protein sequence for *D. melanogaster* (NP_524578.1) was retrieved from the NCBI database. Protein sequences for PHPT1 from *Caenorhabditis elegans* (NP_001369999.1), *Danio rerio* (NP_001003566.1), *Xenopus tropicalis* (XP_002940786.1), *Mus musculus* (NP_083569.1), and *Homo sapiens* were also collected. Multiple sequence alignment of protein was done by ClustalW, the sequence similarity scores were calculated using DNAMAN software.

### Quantification and statistical analysis

Unless otherwise stated, all statistical analyses and graphs were generated using the R language, with results expressed as mean ± SEM and performed by two-tailed Student’s *t*-test. Significance was determined based on *P* -values, with *p* < 0.05 (∗), *p* < 0.01 (∗∗), and *p* < 0.001 (∗∗∗). Exact sample sizes and numbers of biological replicates are indicated in the figure legends.

## Supplemental information

Document S1. Figures S1–S3, Table S1, and Data S1

Data S2. Supplemental Table qPCR raw data, related to Figures 2, 3, and S2
