# Supplementary material for: PHPT1/Ocnus negatively regulates the JAK/STAT pathway to control cell survival
Source: iScience. 2026 Sep 17;29(10):117537. doi: 10.1016/j.isci.2026.117537 (PMC13602238; doi:10.1016/j.isci.2026.117537)

iScience, Volume *29*

Supplemental information

PHPT1/Ocnus negatively regulates the JAK/STAT pathway to control cell survival

Qian Wang, Yue Ren, Meng-Yan Chen, Bin Mao, Xuan Guo, Jian Wan, Ke Liu, Jing-Jing Tong, and Yu-Feng Wang

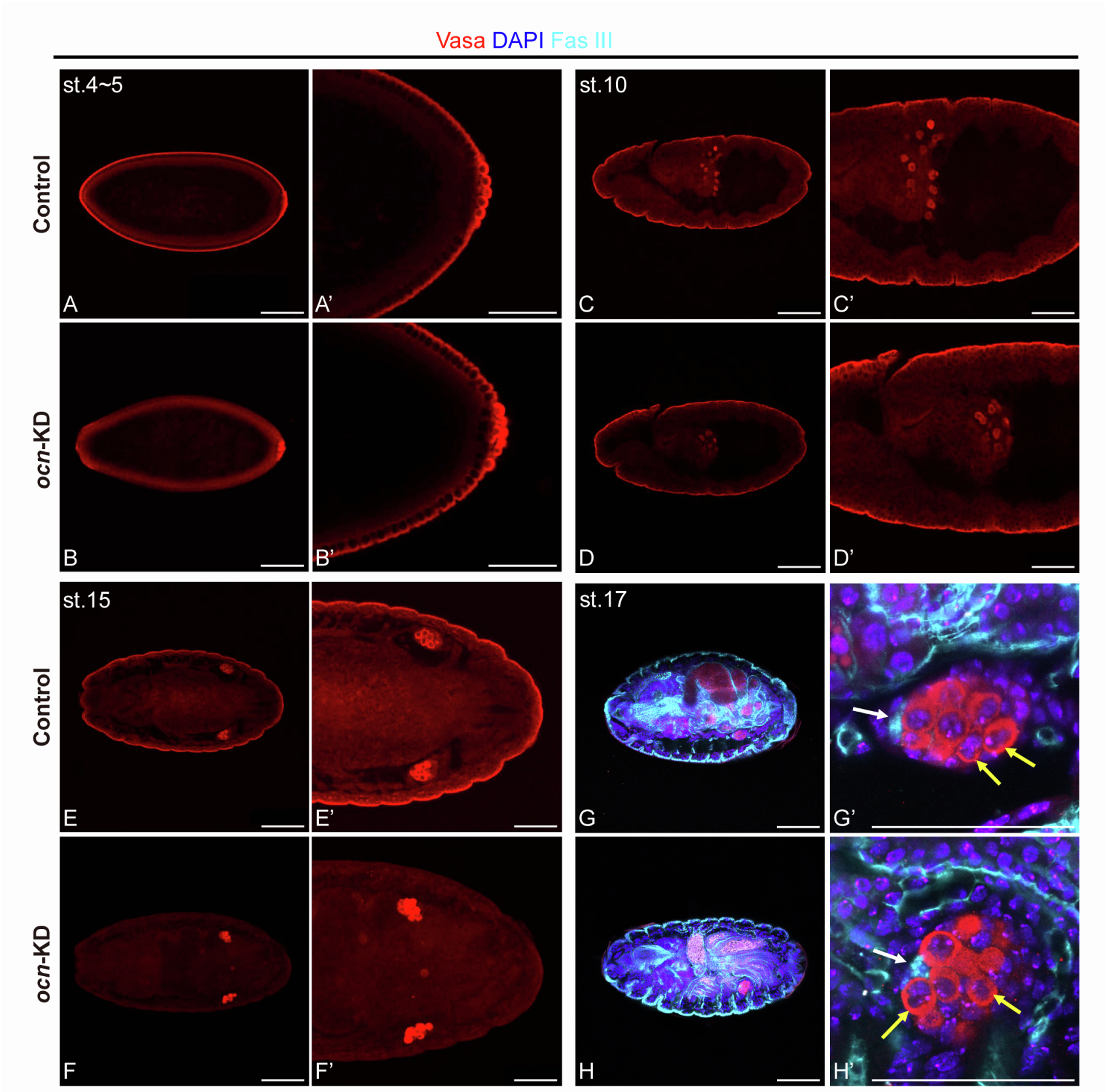

Figure S1. ***ocn*** **knockdown (*ocn*-KD) did not affect the specification and migration of primordial germ cells (PGCs) in the developing embryo of *D. melanogaster,* related to Figure 1.**

Anti-Vasa staining (red) labels PGCs, and DAPI (blue) marks nuclei.

(A and B) The PGCs are specified at the posterior poles (A', B') of the stage 4~5 embryos in both control (A) and *ocn*-KD (B) embryos.

(C and D) At stage 10, the internalized PGCs (C', D') migrate away from the midgut toward the adjacent mesoderm in both control (C) and *ocn*-KD (D) embryos.

(E and F) At stage 15, the germ cells merge and finally compact into round gonads in the posterior (E', F') of both control (E) and *ocn*-KD (F) embryos.

(G and H) Male gonads (white dashed circles) differentiate specific hub cells (Fas III-labeled, cyan; white arrows in G' and H') in both control (G) and *ocn*-KD (H) embryos. Yellow arrows in G' and H' (red) indicate male germ cells.

Representative images are shown from one independent experiment, with a total of 16 embryos analyzed. Scale bars: 100 μm (A-H), 50 μm (A'-H').

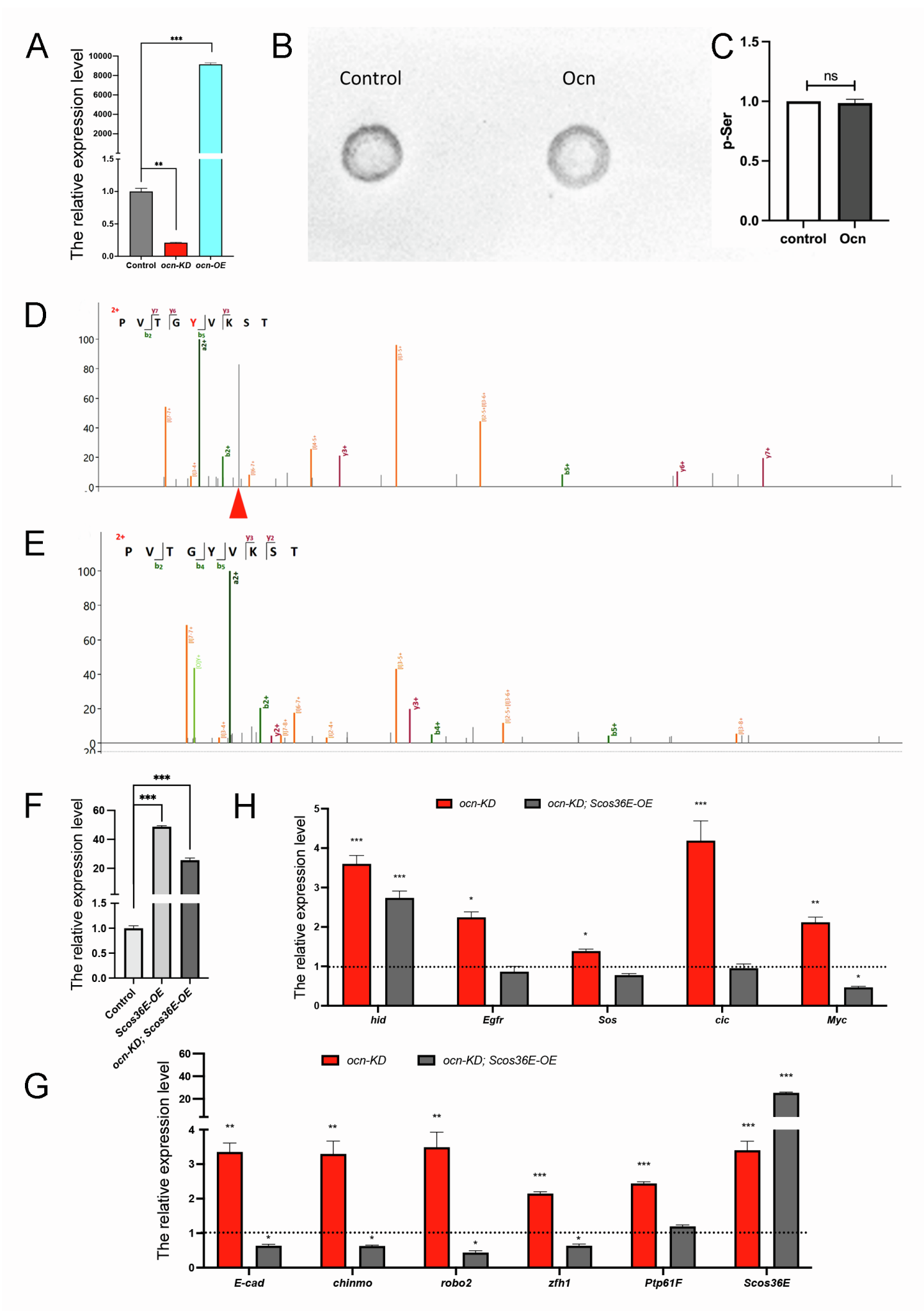

Figure S2. Ocn negatively regulates the JAK/STAT signaling pathway by specifically dephosphorylating the Y711 residue of Stat92E, related to Figures 2 and 3.

(A) RT-qPCR analysis showing relative *ocn* mRNA expression in S2 cells after *ocn*-KD or *ocn*-OE.

(B and C) Dot blot analysis showing the failure of Ocn to dephosphorylate the pS714 peptide of Stat92E. (D and E) MS/MS analysis of the dephosphorylation of STAT at tyrosine (corresponding to Y711 of Stat92E) in the presence of Ocn. The identification of the peptide sequence PVTGYVKST was confirmed by the detection of specific b-ions (green) and y-ions (red). The absence of the phosphorylated fragment ion corresponding to Y711 (E) indicates that dephosphorylation occurred specifically at this residue (red arrow in D).

(F) RT-qPCR analysis showing relative *Scos36E* mRNA expression in S2 cells after *Scos36E*-OE or *ocn*-KD; *Scos36E*-OE.

(G) Overexpression of *Socs36E* partially rescued the transcriptional aberrations of STAT target genes caused by *ocn* depletion.

(H) Overexpression of *Socs36E* inhibited the upregulated expression of pro-apoptotic genes induced by *ocn* depletion.

For RT-qPCR analysis, experiments were performed with a minimum of three biological replicates. Data are shown as mean ± SEM. Student's *t*-test, * P<0.05; ** P<0.01; *** P < 0.001.

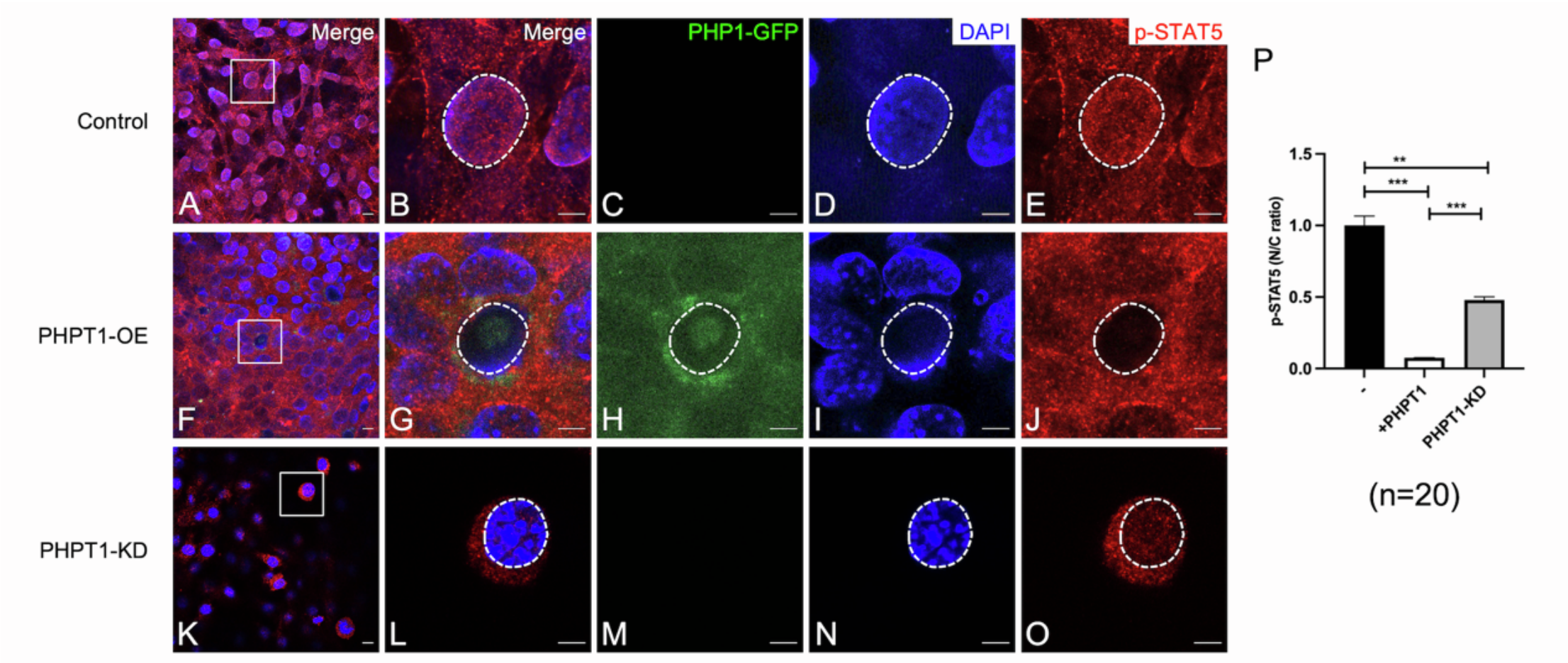

Figure S3. Mouse PHPT1 expression in spermatogenic GC-2 cells influences the subcellular localization of pSTAT, related to Figure 6.

The red fluorescent signal indicates the pSTAT, the green signal represents PHPT1, and the nuclei were stained with DAPI (blue).

(A-E) The subcellular localization of pSTAT in GC-2 cells with the empty vector (Con). (B-E) Magnified views of cells in (A).

(F-J) The subcellular localization of pSTAT in GC-2 cells with the vector carrying mouse PHPT1-GFP (PHPT1-OE). (G-J) Magnified views of cells in (F).

(K-O) The subcellular localization of pSTAT in GC-2 cells with the vector carrying mouse PHPT1-siRNA (PHPT1-KD). (L-O) Magnified views of cells in (K).

Scale bar: 10 μm (A, F, K); 5 μm (B-E, G-J, L-O).

(P) The nucleoplasmic fluorescence signal intensity of pSTAT in PHPT1-expressing cells or knockdown cells, relative to control. n represents the number of cells from two independent biological repeats. The white dashed circle outlines the nucleus. Data are represented as mean ± SEM. Student's *t*-test, $***P < 0.001$.

Table S1 Sequences of the siRNAs and primers used in this study, related to Figure 2 and 3.

Name	Sense (5'-3')	Antisense (5'-3')
NC	UUCUCCGAACGUGUCACGUTT	ACGUGACACGUUCGGAGAATT
ocn siRNA-97	CCUAGAGUUCAGCUUUCUATT	UAGAAAGCUGAACUCUAGGTT
ocn siRNA-300	GGACAAGAAAGUAAUGAAATT	UUUCAUUACUUUCUUGUCCTT
ocn siRNA-386	GGACCAAAUUCCAGCAUUATT	UAAUGCUGGAAUUUGGUCCTT
rp49	CGGTTACGGATCGAACAAGC	CTTGCGCTTCTTGGAGGAGA
E-cad	GAAAATGCCCAGCCCGACG	CATACGATTGGCCGGGTCTC
chinmo	GAGAACACGCCCACAAATGG	GACAGACCTTTGACCTGCAAAC
robo2	TACGGGTTCGCATCGCATAA	AATTCGTCGCGGAGAAATGC
zfh1	GCATCTCACACGAGGACGAG	GTAGGGTCGTTGACCGGAATG
Ptp61F	CGCATGGGCCTCATACAGAC	GCGTCAAGAAAGGTGGGATCG
Socs36E	GTCACAAGTTCAGCTTCGACT	CTCGAAGAACATCACGCAGG
ocnH70A	CCGCCTCCAAGGACGCCGAGGAAATCTTT	GCGTCCTTGGAGGCGGATCCTCTTA
hid	TACCTACTACGCGGGCTACA	AGGTCAATCGCTTGCGTCT
Egfr	GCTGCCCAACGAGAATTTGG	TCCGCGAATGATTTGTAGTTTGG
Sos	GCCAGACGCACCCACATTAC	CGTGGCTTTGTCGCAAGTAG
cic	GGACGCTACTCAGCTATCGG	GGCGCATTGTAGCCGTTTT
Myc	CTTTGACCTGCGCCATCATC	ACATCGATTTCCTCATCGGAAT

Data S1 images

Fig. 2A Co-IP

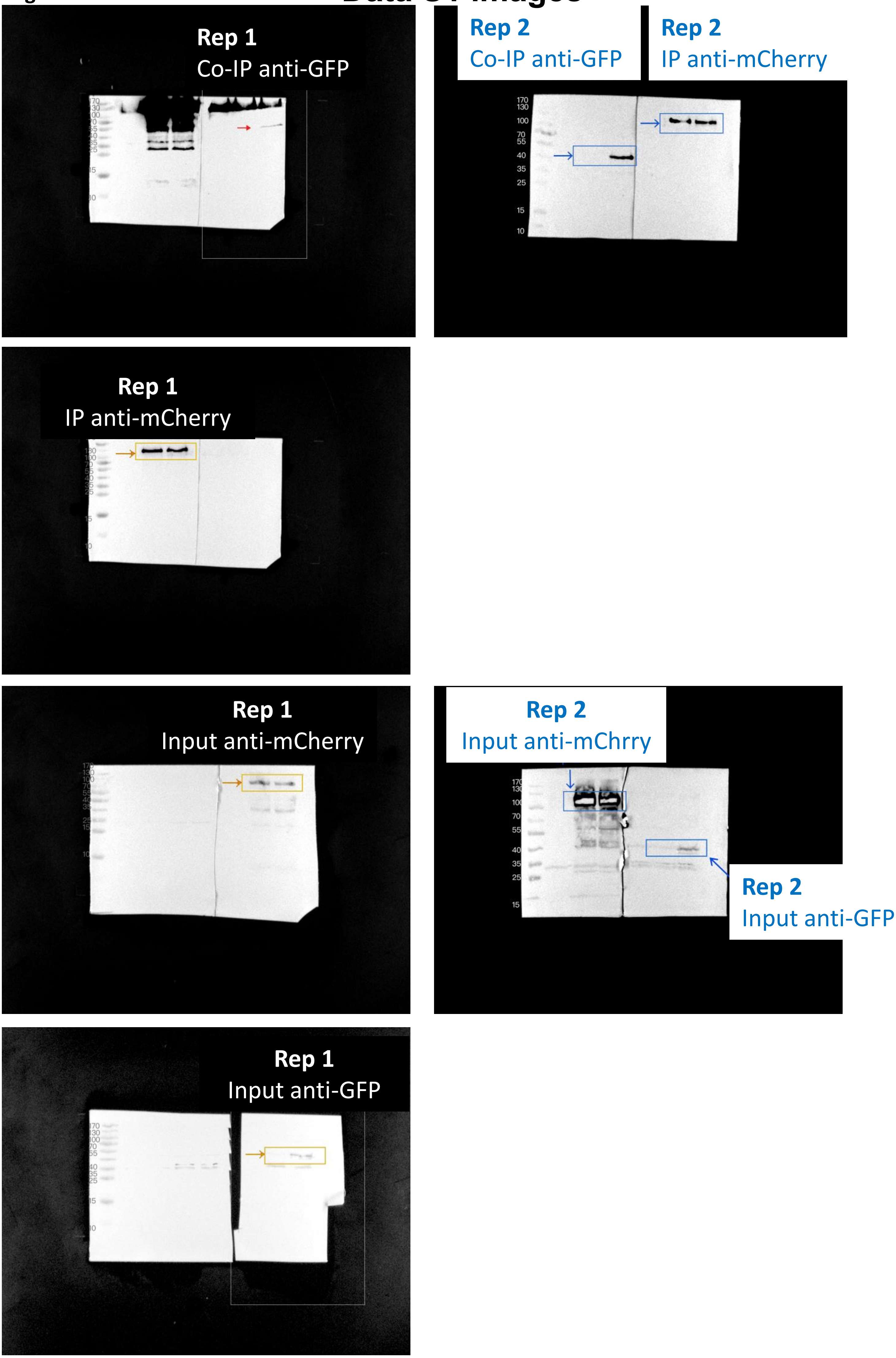

Fig. 2C

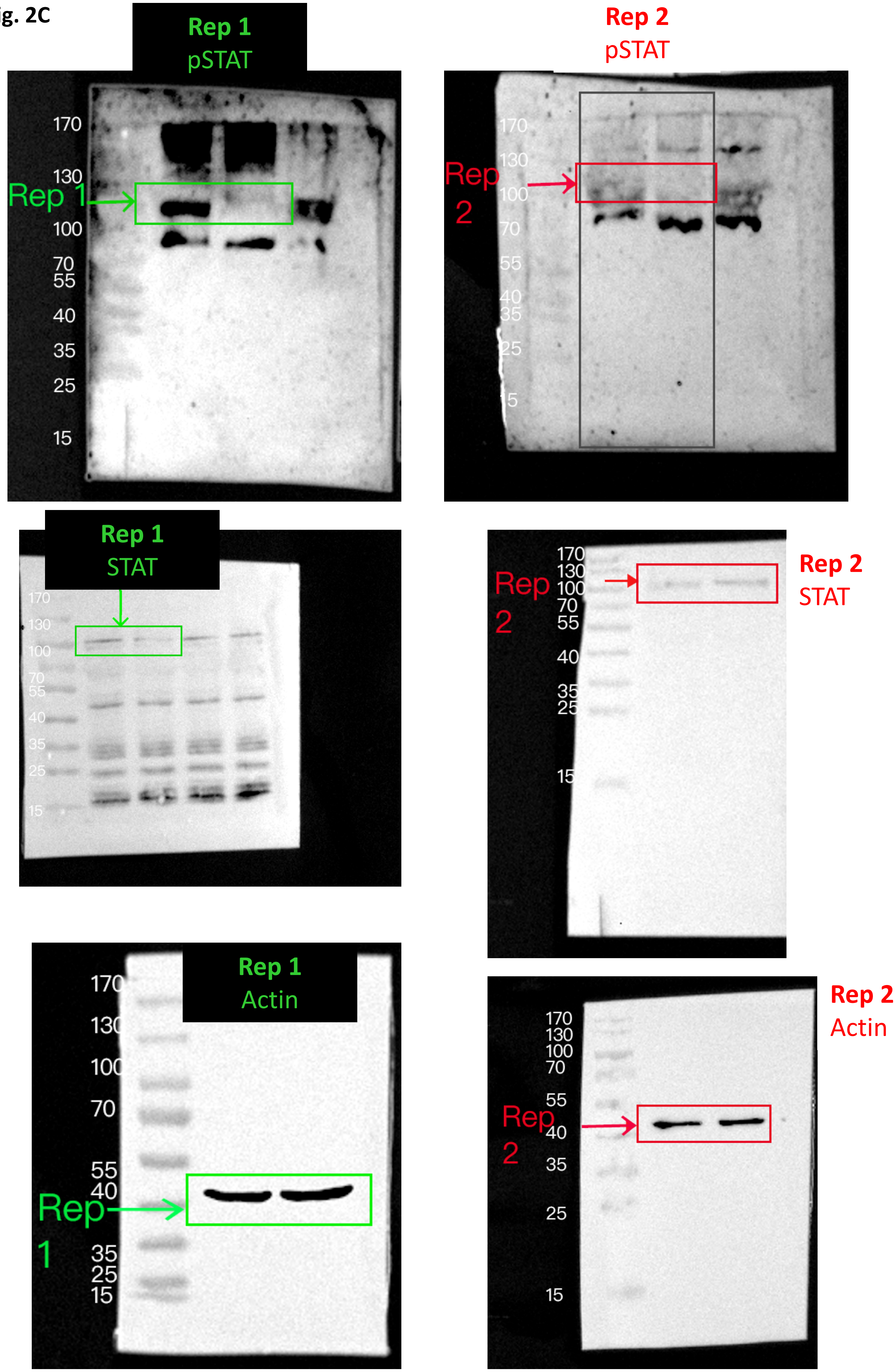

Rep 1
pSTAT
170
130
Rep 1
100
70
55
40
35
25
15
Rep 2
pSTAT
170
130
Rep
2
100
70
55
40
35
25
15
Rep 1
STAT
170
130
100
70
55
40
35
25
15
170
130
Rep
2
100
70
55
40
35
25
15
Rep 2
STAT
Rep 1
Actin
170
130
100
70
55
40
Rep
1
35
25
15
Rep 2
Actin
170
130
100
70
55
Rep
2
40
35
25
15

Fig. 2D

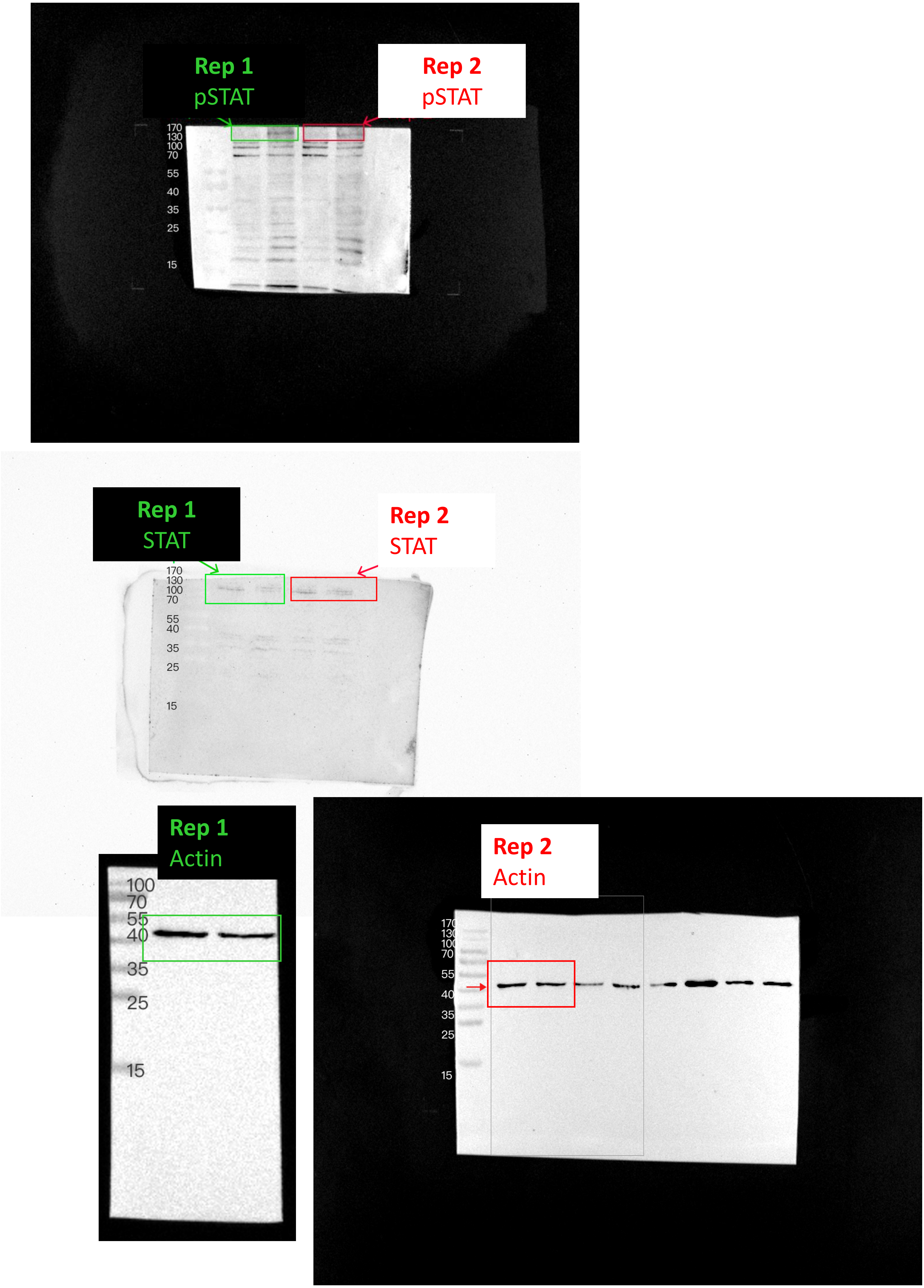
Rep 1
pSTAT
Rep 2
pSTAT
170
130
100
70
55
40
35
25
15
Rep 1
STAT
Rep 2
STAT
170
130
100
70
55
40
35
25
15
Rep 1
Actin
100
70
55
40
35
25
15
Rep 2
Actin
170
130
100
70
55
40
35
25
15

Fig. 2J

Rep 1

Rep 2

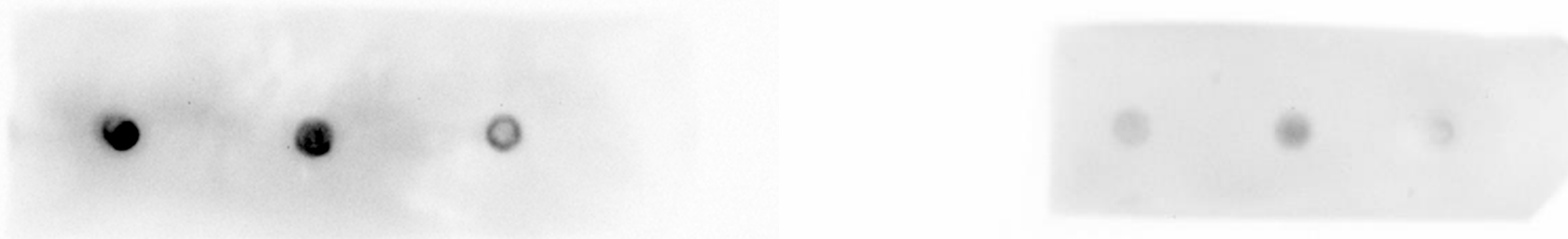

Fig. 2L

Rep 1

Rep 2 and 3

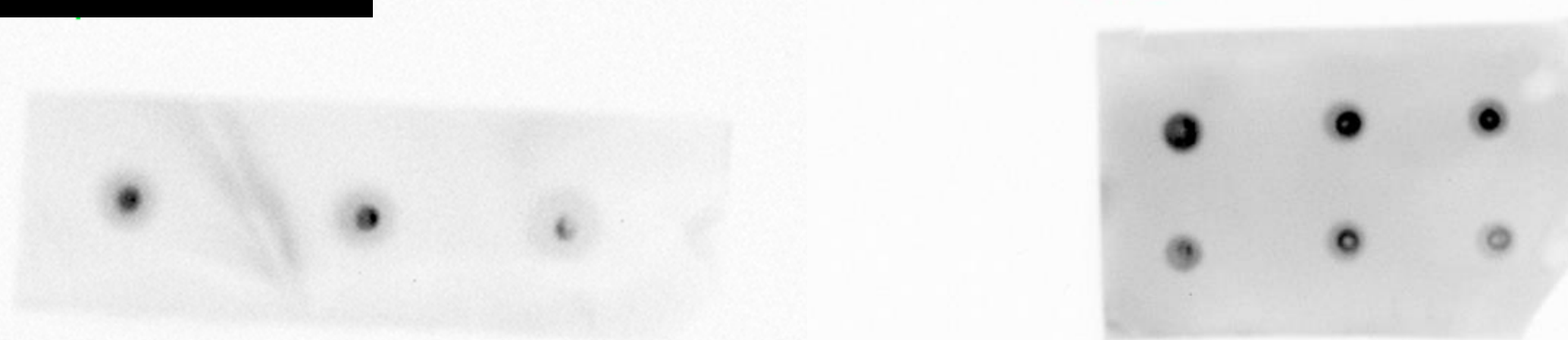

Supplement: Supplementary file 1 [file mmc1.pdf]
